# The RNA repair proteins RtcAB regulate transcription activator RtcR via its CRISPR-associated Rossmann fold domain

**DOI:** 10.1016/j.isci.2022.105425

**Published:** 2022-10-20

**Authors:** Ioly Kotta-Loizou, Maria Grazia Giuliano, Milija Jovanovic, Jorrit Schaefer, Fuzhou Ye, Nan Zhang, Danai Athina Irakleidi, Xiaojiao Liu, Xiaodong Zhang, Martin Buck, Christoph Engl

**Affiliations:** 1 Department of Life Sciences, Faculty of Natural Sciences, Imperial College London, London SW7 2AZ, UK; 2 School of Biological and Behavioural Sciences, Queen Mary University of London, London E1 4NS, UK; 3 Section of Structural Biology, Faculty of Medicine, Imperial College London, London SW7 2AZ, UK; 4 Houston Methodist Research Institute, Houston, TX 77030, USA; 5 College of Food Science and Engineering, Northwest A&F University, Yangling 712100, China

**Keywords:** Molecular biology, Molecular mechanism of gene regulation, Cell biology

## Abstract

CRISPR-associated Rossmann fold (CARF) domain signaling underpins modulation of CRISPR-Cas nucleases; however, the RtcR CARF domain controls expression of two conserved RNA repair enzymes, cyclase RtcA and ligase RtcB. Here, we demonstrate that RtcAB are required for RtcR-dependent transcription activation and directly bind to RtcR CARF. RtcAB catalytic activity is not required for complex formation with CARF, but is essential yet not sufficient for RtcRAB-dependent transcription activation, implying the need for an additional RNA repair-dependent activating signal. This signal differs from oligoadenylates, a known ligand of CARF domains, and instead appears to originate from the translation apparatus: RtcB repairs a tmRNA that rescues stalled ribosomes and increases translation elongation speed. Taken together, our data provide evidence for an expanded range for CARF domain signaling, including the first evidence of its control via *in trans* protein-protein interactions, and a feed-forward mechanism to regulate RNA repair required for a functioning translation apparatus.

## Highlights

• RtcR physically interacts with RtcB and RtcA via its regulatory CARF domain
• RtcR-RtcBA interactions positively control *rtcBA* expression
• The transfer-messenger RNA *ssrA* is a target of *rtc* inducing stress
• RtcB impacts the speed of translation elongation

## Graphical abstract

**Figure:**
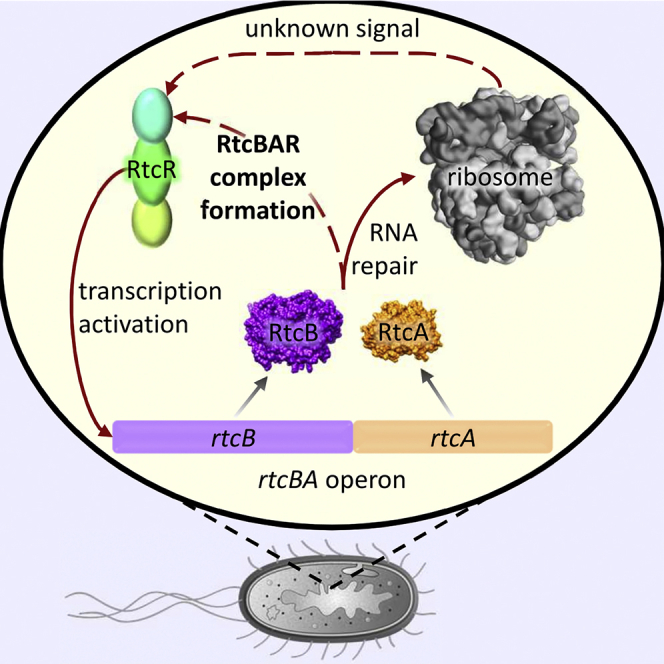

## Introduction

Clustered, regularly interspaced short palindromic repeats (CRISPR) and the associated Cas proteins are responsible for acquired immunity against foreign genetic elements in prokaryotes^1^^,^^2^^,^^3^ and CRISPR-associated Rossmann-fold (CARF) domain signaling is responsible for the modulation of CRISPR-Cas systems: oligoadenylate synthetases generate oligoadenylate nucleotides when interacting with their nucleic acid targets and subsequent oligoadenylate binding by CARF domains regulates downstream nucleases and other effectors in a novel signaling system to degrade foreign nucleic acids.^4^^,^^5^ In contrast to the regulation of nucleic acid degradation, the CARF domain containing RtcR protein of diverse bacteria activates expression of the nucleic acid repair enzymes, RNA cyclase RtcA and RNA ligase RtcB.^6^^,^^7^

RtcA and RtcB are conserved in eukaryotes, archaea and bacteria. RtcA converts RNA 3′-P ends, and to a lesser extent 2′-P ends, to a 2′,3′-cyclic P form,^7^^,^^8^ such that RtcB can then ligate it to a 5′-OH terminated RNA in a GTP-dependent manner to form a new 5′-3′ linkage.^6^ In higher eukaryotes, RtcB has been strongly implicated in several important cellular processes, including tRNA maturation as part of the tRNA-splicing ligase complex,^9^^,^^10^ the unfolded protein response,^11^ endoplasmic reticulum stress^12^ and neuron regeneration.^13^ In contrast, relationships between cell function and RtcA are not well defined.^14^ Bacterial RtcB and RtcA have been studied in structural and biochemical detail; however, their physiological roles are largely unknown. Potential areas of their importance include ribosome and tRNA integrity, together with cellular responses to ribotoxins, antibiotics and oxidative stress.^15^^,^^16^^,^^17^^,^^18^

**Figure 1:**
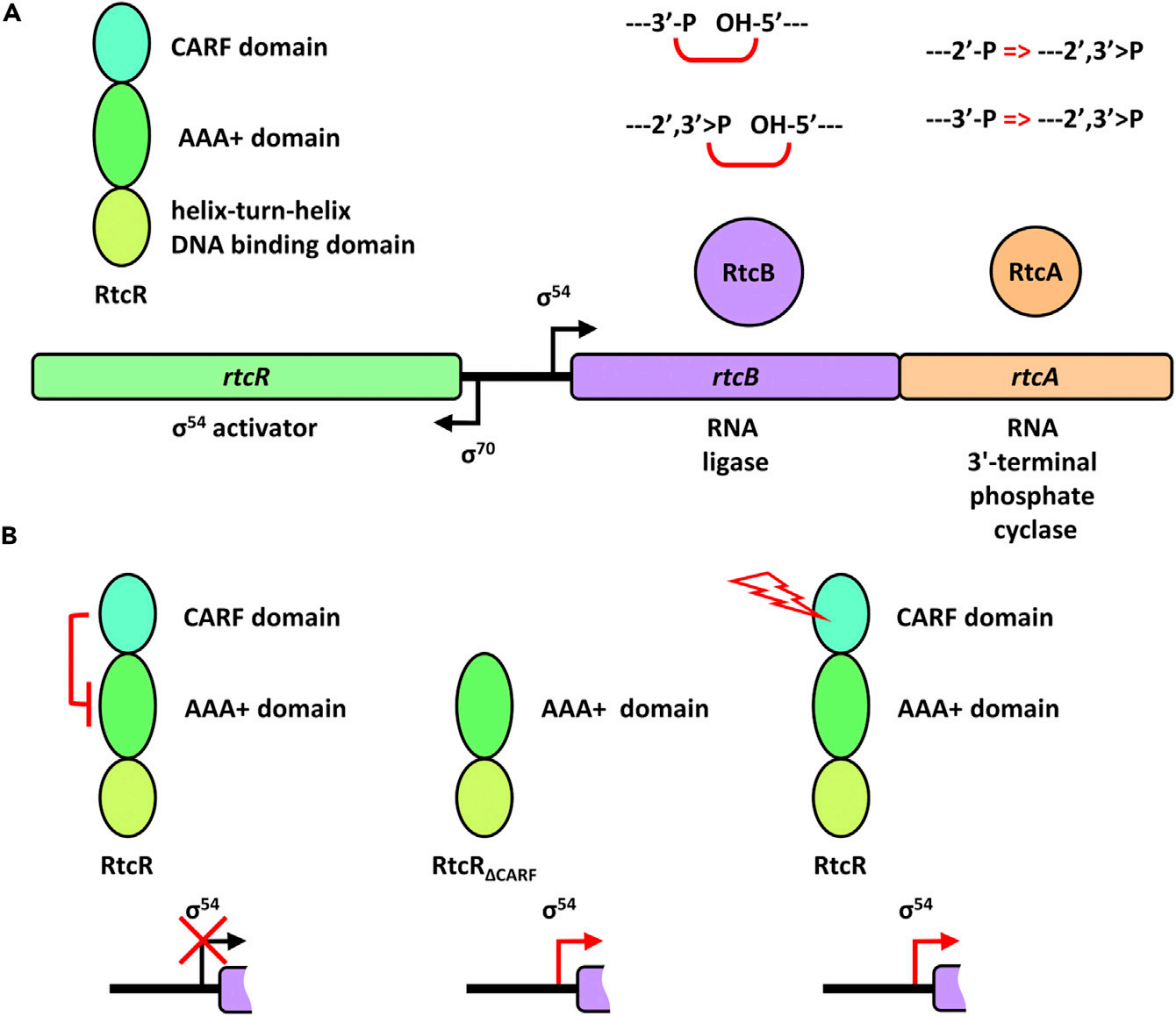
Schematic representation of the Rtc system (A) The *rtcBA* operon is under the control of the sigma 54 dependent *rtcBA* promoter whose activator is encoded by the oppositely oriented *rtcR* gene. RtcA is an RNA 3′-terminal phosphate cyclase that converts 2′-P and 3′-P ends into 2′,3′-cyclic P ends. RtcB is an RNA ligase that ligates 3′-P and 2′,3′-cyclic P ends to 5′-OH ends. (B) The sigma 54 transcriptional activator RtcR has three functional domains including a N-terminal, regulatory CARF domain. Under normal conditions, the CARF domain represses the AAA + domain so that no transcription of *rtcBA* takes place from the *rtcBA* promoter (left). Conversely, RtcR mutants lacking the CARF domain are constitutively active (middle). Under stress, an unknown signal ligand interferes with the CARF domain mediated repression of the AAA + domain and the *rtcBA* operon is expressed (right).

In the model organism *Escherichia coli*, RtcA and RtcB are encoded by the *rtcBA* operon.^8^ Notably, *rtcBA* operons are conserved in diverse bacteria.^7^ The expression of the *E*. *coli rtcBA* operon relies on a σ^54^–dependent promoter and the transcriptional activator RtcR, a bacterial enhancer-binding protein whose central AAA + domain interacts with the σ^54^ RNA polymerase holoenzyme and activates expression of *rtcBA* (Figure 1A). Similar to regulation seen in CRISPR/Cas systems, the CARF domain of RtcR has inhibitory function repressing RtcR activity (Figure 1B).^8^ In this work we study RtcR-dependent activation of *rtcBA* transcription and reveal the presence of a RtcRAB regulatory complex mediated via the RtcR CARF domain. This is the first evidence of a CARF domain regulation via *in trans* protein-protein interaction and a novel protein complex in which RtcB is found.^10^ We also identify novel RNA targets of RtcB action and propose how they may serve as signal molecules acting on the RtcR CARF domain.

## Results

### The *rtcBA* locus is essential for RtcR activation

The Rtc system is induced by diverse stimuli in bacteria, some activating *rtcBA* expression independently of RtcR, as shown previously in *S*. *enterica*,^17^ likely due to global impact on mRNA synthesis and stability, or the use of alternative promoters. Here we sought to establish which Rtc-inducing conditions relay activating signals via RtcR and its CARF domain in *E*. *coli*, and whether the products of its action, RtcB and RtcA, are also involved in signaling. This is the case for other *E*. *coli* operons modulated by bacterial enhancer-binding proteins, such as the phage shock protein (Psp) membrane stress response: the dual functional PspA protein acts as both the effector of the Psp response and the negative regulator of its transcriptional activator PspF.^19^^,^^20^

To this end, genetic determinants leading to Rtc induction were identified and assessed for their ability to increase activity of the *rtcBA* promoter in the absence of *rtc* genes (Table 1). Gene deletion mutants of the glutathione reductase *gor* (Table S1 and Figures S1A and S1C), the stress-induced *yobF* (Table S1 and Figures S1A and S1D) and the tRNA deacetylase *ybaK* (Table S1 and Figure S1E) were previously shown to upregulate *rtcBA* promoter activity.^15^ Here, we selected KEIO deletion mutants whose protein function could potentially be associated with RNA repair and the Rtc system based on findings presented in Engl et al., (2016)^15^ and Kurasz et al., (2018).^17^ Genes linked to RNA modifications, the ribosome, or oxidative stress were considered likely candidates for Rtc induction and were tested for their ability to upregulate *rtcBA* promoter activity via β-galactosidase assays with cells harboring a plasmid-encoded P_*rtcBA*_-*lacZ* reporter. In parallel, we screened the ASKA library to identify genes whose over-expression leads to Rtc induction. Deletion mutants of RNA helicase *srmB* (Table S1 and Figure S1A), ribonuclease *mazF* (Table S1 and Figures S1A, S1F, and S1G), endoribonuclease *rnhA* (Table S1 and Figure S1H), RNA pyrophosphohydrolase *rppH* (Table S1 and Figure S1I),and RNA binding protein *hfq* (Table S1 and Figures S1J and S1K), together with strains over-expressing transcription antiterminator *rof* (Table S2 and Figures S1L and S1M) and histidine kinase *yedV* (Table S2 and Figures S1N and S1O) led to significant upregulation of the *rtcBA* promoter activity as compared to the wild-type. Of interest, the *hfq* gene deletion caused induction of the Rtc system in rich media (Table S1 and Figure S1K) and repression in minimal media (Table S1, Figure S1J), suggesting antagonistic roles of *hfq* consistent with divergent physiological responses of *E*. *coli* under different growth conditions.^21^ RT-qPCR (Tables S1 and S2, and Figure S1B) confirmed the reporter assays, demonstrating increased abundance of the chromosomally derived *rtcBA* mRNAs concomitant with the upregulated *rtcBA* promoter activity.

**Table 1:** Genes and gene deletion mutants modulating the expression of the Rtc system

Name	Description	Effect	Dependent on		
			RtcR	RtcB	RtcA
*ΔahpC*	Peroxidase; forms alkyl hydroperoxide reductase with peroxiredoxin reductase *ahpF* [AhpC]_10_[AhpF]_2_	↑	YES	YES	YES
*Δgor*^a^	GSH oxidoreductase: in operon with 23S rRNA methyltransferase	↑	YES	YES	NO
*Δhfq*	RNA binding protein: regulation of the activity of small RNA species	↑/↓	NO	–	–
*ΔmazF*	Ribonuclease; toxin component of a toxin-antitoxin system	↑	YES	YES	YES
*ΔrnhA*^b^	Endoribonuclease (RNase H); digestion of RNA in DNA-RNA hybrids	↑	NO	–	–
*ΔrppH*	RNA pyrophosphohydrolase; hydrolysis of 5′-triphosphate mRNA end, prior to degradation by RNase E	↑	NO	–	–
*ΔsrmB*	RNA helicase; assembly of the 50S ribosomal subunit	↑	–	–	–
*ΔybaK*^a^	Cys-tRNAPro and Cys-tRNACys deacylase; tRNA editing	↑	YES	YES	YES
*ΔyobF*^a^	stress-induced peptide; in operon with ribosome-associated CspC	↑	YES	YES	NO
*rof*	transcription antiterminator; interaction with Rho terminator protein	↑	NO	–	–
*yedV*	histidine kinase; in operon with yedW which confers drug resistance	↑	NO	–	–
*yheS*	ABC-F protein; mediation of antibiotic resistance via ribosomal protection	↓	–	–	–

a Originally identified by Engl et al. (2016).^15^

b Also identified in *Salmonella enterica* serovar Typhimurium by Hughes et al. (2020).^18^

We next assessed the requirement of *rtcR*, *rtcB* and *rtcA* for transcriptional activation of *rtcBA* expression under these inducing conditions. RtcR-dependent induction of *rtcBA* was only observed in the *gor*, *yobF*, *ybaK* and *mazF* mutants (Table S1 and Figures S1C–S1F). Induction of *rtcBA* in the *rof* mutant was RtcR-dependent in rich medium but RtcR-independent in minimal medium. The phenotypes for the *hfq* and *rof* mutants therefore illustrate that the Rtc system is sensitive to the multilevel effects of external growth conditions. This is consistent with its role in supporting essential basic cellular processes such as translation which needs to occur in response to a wide range of growth conditions. Remarkably, RtcR-dependent transcriptional activation of *rtcBA* expression in those mutants strictly required the presence of a catalytically active form of RtcB (Table S1 and Figures S1C–S1F), suggesting that generation of the *rtcBA* inducing signal which acts on RtcR requires the ligase activity of RtcB. In contrast, the requirement for *rtcA* was dependent on the inducing conditions and was only observed in the *ybaK* and *mazF* mutants (Table S1 and Figures S1E and S1F).

In summary, our data suggest that regulated induction of the Rtc system has novel interdependencies and relies to varying degrees not only on the action of the transcriptional activator RtcR but also on the actions of RtcB and RtcA. These interdependencies create a feed forward mechanism for *rtcBA* expression when RtcR activates the *rtcBA* promoter.

### The RtcRAB proteins interact

**Figure 2:**
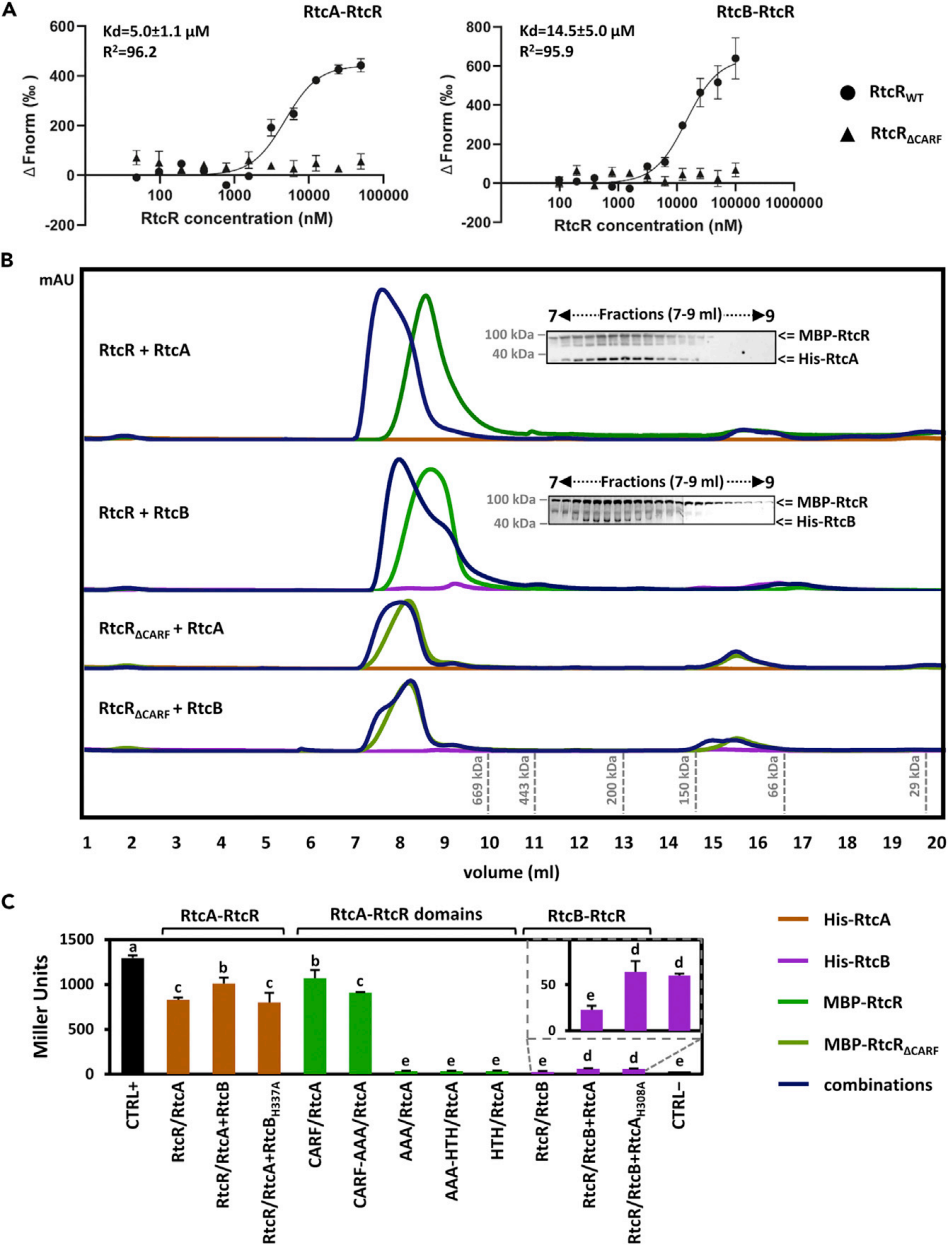
The RtcRAB proteins interact via the RtcR CARF domain (A and B) Rtc proteins were overexpressed and purified (Figure S2A), and their physical interactions were investigated using (A) MST and (B) gel filtration chromatography. (A) Changes in thermophoresis (y axis of graph) of fluorescently labeled RtcR_WT_ and RtcR_ΔCARF_ was used to quantify their binding to RtcA or RtcB in titration experiments. RtcR_WT_, but not RtcR_ΔCARF_, interacts *in vitro* with RtcA (left) and RtcB (right), as shown by MST. The dissociation constant (Kd) of RtcA and RtcB with RtcR is in the μM range. (B) Alterations in the fractionation and apparent molecular weight of RtcR_WT_ and RtcR_ΔCARF_ were used to show their complex formation with RtcA or RtcB. The peak of MBP-RtcR, but not MBP-RtcR_ΔCARF_, potentially forming a hexamer, shifts towards a higher molecular weight in the presence of His-RtcA and His-RtcB, as shown by gel filtration chromatography. Co-localization of MBP-RtcR with His-RtcA and His-RtcB was confirmed with SDS-PAGE and immunoblotting. The proteins were present at equimolar concentrations (20 μM). (C) Pairwise combinations of N-terminal and C-terminal T18/T25 fusions between full length or truncated Rtc proteins (Figures S2B–S2D) were assessed for *in vivo* physical interactions as indicated by elevated β-galactosidase activity in bacterial 2-hybrid assays. RtcR interacts *in vivo* with RtcA via its CARF domain, as shown by bacterial two-hybrid analysis (*N* = 3). In trans RtcA or RtcB influences the interactions of RtcR with respectively RtcB and RtcA, as shown by bacterial three-hybrid analysis (*N* = 3). Data are shown as mean and error bars represent standard deviation from the mean. *N* represents total number of independent biological replicates, with 3 technical replicates each. ANOVA ∗ p-value < 0.05; ∗∗ p-value < 0.01; ∗∗∗ p-value < 0.001; ∗∗∗∗ p-value < 0.0001.

Having established that an intact *rtcBA* locus is essential for full RtcR-dependent transcription activation, we tested whether RtcA and RtcB directly interact with RtcR. Such regulatory complexes are involved in other σ^54^-dependent adaptive strategies of bacteria, such as PspA-PspF and HrpV-HrpRS that control respectively the transcription of a membrane stress response in *E*. *coli*^22^ and virulence factor secretion in the plant pathogen *Pseudomonas syringae*.^23^ We investigated the physical interactions between the Rtc proteins (Figure S2) through a combination of *in vitro* and *in vivo* methods, using microscale thermophoresis (MST; Figure 2A), gel filtration chromatography (Figure 2B), bacterial two-hybrid analysis (Figures 2C and S3) and protein crosslinking (Text S1 and Figure S4).

For MST and gel filtration chromatography, N-terminally tagged Rtc proteins (Figure S2A) were over-expressed and purified using affinity chromatography. Changes in thermophoresis or the apparent molecular weight of RtcR in the presence of RtcA or RtcB were interpreted as evidence of direct physical interactions between the purified proteins. Pairwise binding experiments via MST showed that purified RtcR physically interacts with both RtcA and RtcB *in vitro* (Figure 2A). The dissociation constant (Κd value) of RtcR for RtcA was approximately 5 μM, suggesting that the interaction between the two proteins is moderately weak and hence transient. The interaction between RtcR and RtcB was even weaker with a dissociation constant of 14.5 μΜ (Figure 2A). Such weak and transient interactions are often observed within signaling pathways, allowing for readily reversible regulatory outputs in response to dynamic signal input and as such they enable fast and balanced adaptation under fluctuating conditions.^24^ Gel filtration confirmed the direct interaction of RtcR with RtcA and RtcB *in vitro* (Figure 2B). The elution profile of MBP-RtcR (103 kDa as a monomer) further showed that RtcR self-assembles into apparent hexamers, heptamers or other higher order complexes (Figure 2B), similar to other AAA + transcription activators such as PspF.^25^ The profile shifted to higher molecular weight species comprising both interaction partners (Figure 2B) when MBP-RtcR was mixed together with either His-RtcA or His-RtcB. No evidence for an interaction was detected between His-RtcB and His-RtcA.

We next performed a bacterial two-hybrid assay that reveals protein-protein interactions via a simple *lacZ* reporter gene readout to test whether RtcR-RtcA-RtcB interactions also occur *in vivo*. N-terminal or C-terminal fusions of Rtc proteins with the T18 or T25 domains of adenylate cyclase (Figure S2B) were constructed and introduced as pairs into *E*. *coli* cells; interaction between the protein pairs tested would result in functional complementation between T18 and T25 and subsequent cAMP-dependent expression of a chromosomal *lacZ* reporter gene, which was quantified using β-galactosidase assays. Each protein pair tested was considered to interact if the β-galactosidase assay output was significantly higher than the T18 and T25 domains on their own. The self-association of RtcR as well as the interaction between RtcR and RtcA but not between RtcR and RtcB was confirmed (Figures 2B, S3A, S3C, and S3D). In addition, RtcA did specifically interact with RtcR but not with other AAA + transcription activators such as HrpS (Figures S2D and S3B). In further agreement with the *in vitro* experiments, we did not detect an *in vivo* interaction between RtcA and RtcB (Figures 2B and S3A). Note that the *in vitro* interaction between RtcR and RtcA was moderately weak, whereas the interaction between RtcR and RtcB was even weaker, as shown by MST (Figure 2A). We therefore hypothesized that the pairwise interactions might be strengthened in presence of the third Rtc protein in a tripartite two-hybrid assay, instead of a traditional two-hybrid assay that only tests pairwise interaction. Indeed, ectopic expression of RtcB *in trans* from a pBAD18 cm plasmid significantly affected and almost always increased the interaction of RtcR with RtcA, whereas an interaction between RtcR with RtcB was detectable in the presence of RtcA (Figures 2C, S3C, and S3D). More specifically, out of the six pairs tested and deemed functional (pairs including the RtcR-T18 fusion were never observed to be functional), presence of RtcB significantly increased the RtcR-RtcA interaction in four cases and decreased the interaction in one case (Figure S3C). Similarly, presence of RtcA significantly increased the RtcR-RtcB interaction in four cases (Figure S3D). Notably, catalytic mutants of RtcA and RtcB showed the same outcome in tripartite two-hybrid assays as their wild-type versions; therefore, neither RNA end healing by RtcA nor RNA end sealing by RtcB is required for the formation of the RtcRAB complex (Figures 2C, S3C, and S3D).

In summary, we conclude that RtcRAB together can form a transient complex *in vivo* in which RtcA and RtcB mutually facilitate their attachment to RtcR without directly binding to each other. We speculate that the physical interactions between RtcR, RtcA and RtcB provide a basis by which RtcA and RtcB impact on the ability of RtcR to activate the *rtcBA* promoter.

### RtcRAB complex formation is mediated via the RtcR CARF domain

We next sought to identify the determinants for the formation of the RtcRAB complex. We therefore subjected truncated versions of RtcR (Figures S2A and S2C) to the same *in vitro* and *in vivo* assays as full-length RtcR earlier. Lack of the CARF domain (RtcR_ΔCARF_) prevented the physical interaction of RtcR with both RtcA and RtcB, as shown by MST (Figure 2A), gel filtration chromatography (Figure 2B), bacterial two-hybrid analysis (Figures 2C and S3F) and protein crosslinking (Text S1 and Figure S4). Moreover, the CARF domain on its own could bind to RtcA (Figure S3G) and RtcB (Figure S3H); the latter interaction was abolished in the presence of RtcA (Figure S3H), as shown by two-hybrid assays. This contrasts the observation with full-length RtcR and indicates that domains of RtcR other than CARF help stabilizing the RtcRAB complex. RtcR could still self-assemble in absence of its CARF domain (Figure S3E), however it appears to form larger oligomers during gel filtration chromatography than full-length RtcR (Figure 2B). We speculate that the absence of the N-terminal regulatory domain, which traditionally controls self-assembly of AAA + enhancer binding proteins^26^ (Bush & Dixon, 2012), leads to increased self-assembly of the RtcR protein.

In summary, a range of independent *in vitro* and *in vivo* protein-protein interaction data provide clear evidence that the direct physical interaction of RtcA and RtcB with RtcR occur through its CARF domain. This novel tripartite protein complex is essential for signaling and transcription regulation.

### RtcA and RtcB are not sufficient for RtcR-dependent transcription activation in vitro

We established an *in vitro* transcription assay using purified components to measure RtcR activity at a test promoter to determine whether protein-protein interactions between RtcR, RtcA and RtcB are sufficient for RtcR-dependent activation of the σ^54^–RNA polymerase holoenzyme and subsequent *rtcBA* expression. When RtcR is present in an active form, i.e., either lacking its CARF domain or potentially bound by an activating ligand, the σ^54^ RNA polymerase holoenzyme adds radioactive ribonucleotides to a primer dinucleotide (at −1,+1 transcription initiation site) resulting in the specific labeled template dependent synthesis of a short primed (sp) RNA molecule which is then visualized by urea-PAGE. The wild-type *rtcBA* promoter proved to be very weak *in vitro* and so a hybrid test promoter containing the *E*. *coli rtcBA* upstream activation sequence (UAS) for RtcR binding, the *rtc* integration host factor (IHF) binding site, and the *Sinorhizobium meliloti nifH* −24/-12 DNA σ^54^ binding site (instead of that from the *rtcBA* promoter) was constructed for *in vitro* transcription assays.

**Figure 3:**
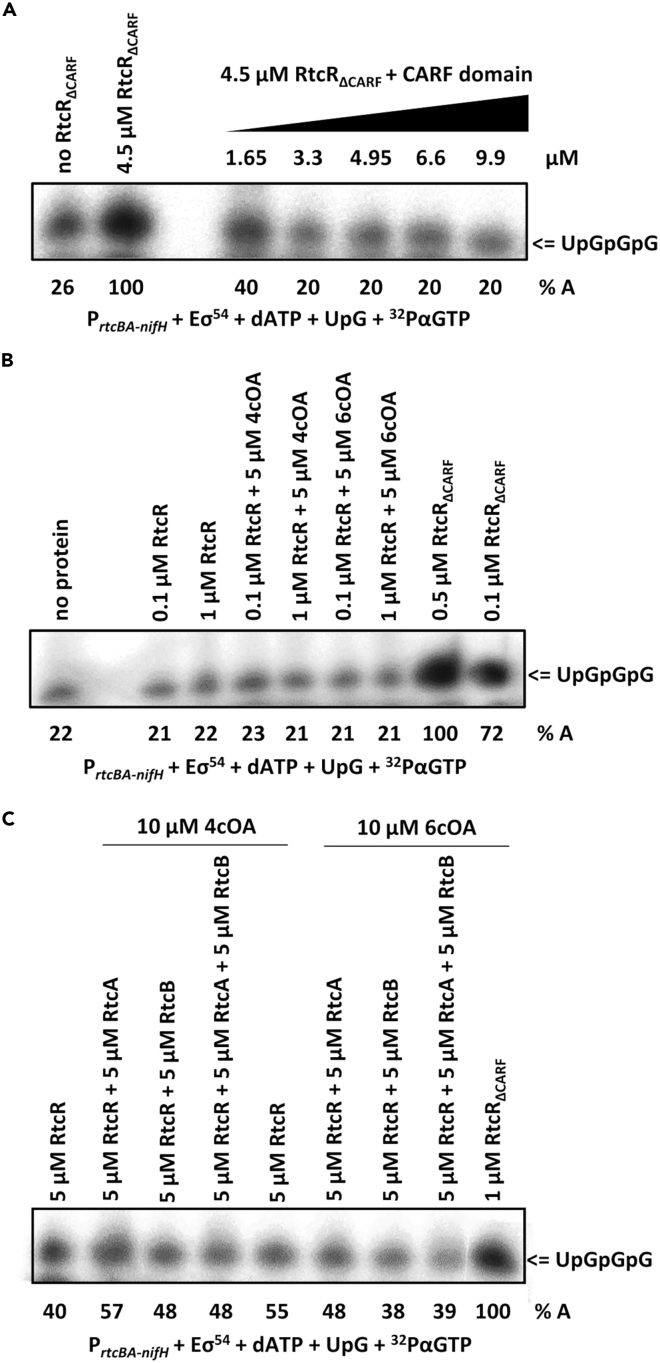
The RtcR CARF domain is repressive, and RtcR is not activated by RtcAB or oligoadenylates *in vitro* The transcriptional activity of purified RtcR and its CARF deletion variant was measured in an *in vitro* transcription assay leading to synthesis of radioactive RNA UpGpGpG following extension of UpG by addition of radioactive GTP from the super-coiled P_*rtcBA-nifH*_ hybrid promoter template. (A–C) The RtcR_ΔCARF_ variant, in the presence of in trans CARF domain, (B) full length RtcR and cyclic tetra/hexa (4/6) adenylates (cOA), or (C) full length RtcR, RtcA and/or RtcB and cOA. The UpGpGpG was separated from unincorporated GTP by electrophoresis on a 20% (w/v) urea-PAG. In panels (A–C), the %A is percentage of transcription activity versus that seen with the RtcR_ΔCARF_ variant.

Purified RtcR_ΔCARF_ was constitutively active for stimulating transcription *in vitro* (Figures 3 and S5) whereas the full length RtcR lacked such activity, consistent with the N-terminal CARF domain having a repressive effect on RtcR activity.^8^ In addition, when added *in trans* the CARF domain was able to repress the activity of RtcR_ΔCARF_ (Figure 3A), illustrating that the CARF domain does not have to be contiguous with the RtcR central catalytic domain to repress transcription activation by RtcR. The full-length purified RtcR was unable to activate transcription from the test promoter (i) alone, (ii) in the presence of purified RtcA and/or RtcB (Figure S5A), and (iii) under a range of conditions chosen to reflect potential inducing conditions *in vivo* (Table S3). The latter included substitution of magnesium by manganese, which is an essential co-factor for RtcB (Figure S5B).

Thus, although essential *in vivo*, RtcA and RtcB are not sufficient for transcriptional activation by RtcR *in vitro*. This is consistent with an additional requirement of ligand binding to the RtcR CARF domain to release its inhibitory effect on RtcR activity. Consequently, we next added a range of potential ligands to our *in vitro* assays to assess their ability to stimulate RtcR-dependent transcription activation.

### Oligoadenylates are not a signal for *RtcR-dependent transcription activation*

CARF domains responsible for the modulation of CRISPR-Cas systems are activated by cyclic oligoadenylate nucleotides (cOAs) generated by the Palm domain of oligoadenylate synthetases when interacting with their nucleic acid targets; subsequent cOA binding by CARF domains regulates downstream nucleases and other effectors.^4^^,^^5^ We tested in our *in vitro* transcription assay whether RtcR responds to the same ligands (cyclic or linear oligoadenylates) that stimulate the activity of other CARF-dependent protein systems.^4^^,^^5^ Purified RtcR bound cOAs very weakly *in vitro* and neither cyclic nor linear oligoadenylates were sufficient to alleviate the repressive effect of the CARF domain on RtcR activity *in vitro* (Figures 3B and 3C), suggesting that they are unlikely the true physiological ligands of RtcR.

Recently, Hughes et al. (2020)^18^ reported that the RtcR CARF domain in *Salmonella enterica* serovar Typhimurium binds tRNA fragments and proposed that this interaction may lead to RtcR activation. We explored this possibility for our *E*. *coli* Rtc system by performing an electrophoretic mobility shift assay (EMSA) using purified *E*. *coli* RtcR protein and tRNA fragments and by adding similar tRNA fragments to the *in vitro* transcription assay. Fragments of tRNAs stably bound the RtcR CARF domain, consistent with the findings of Hughes et al. (2020),^18^ but not the full length RtcR, as shown by EMSA (Figure S5C) and they were not sufficient to activate RtcR for *in vitro* transcription.

In summary, we conclude that any small molecule ligand that is recognized by the RtcR CARF domain acts as the activating signal for RtcR-dependent transcription activation and is novel as compared to other CARF-dependent proteins; we further propose that it is an RNA that contains a 2′,3′-cyclic P at its 3′ terminus based on the conservation of the CARF domain and its ability to recognize cyclic nucleic acid molecules.

### The Rtc system is induced under conditions of elevated *in vivo* hydrogen peroxide formation

Induction of the Rtc system in cells lacking the glutathione reductase *gor* (Table 1) links *rtcBA* expression to oxidative stress. We further investigated the basis of this observation using the *E*. *coli* hydroperoxidase mutant Hpx-, a MG1655 strain reported to lack the genes encoding catalases KatE and KatG and the alkyl hydroperoxide reductase components AhpC and AhpF (Text S2). Hpx- is therefore unable to detoxify H_2_O_2_ species within the cell.^27^

**Figure 4:**
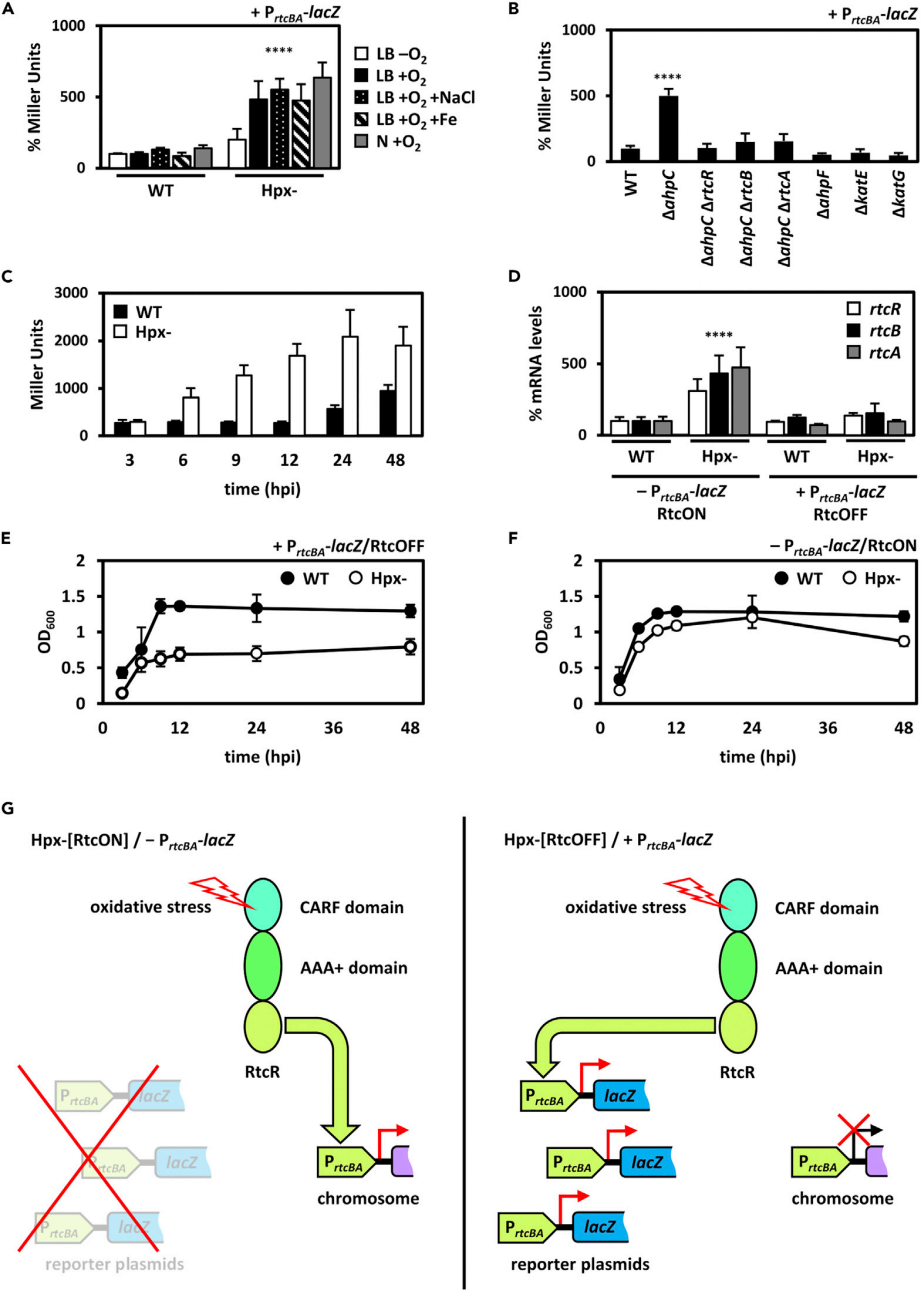
The Rtc system is induced in the Hpx- strain The transcriptional activation of the Rtc system in the Hpx- strain was assessed using β-galactosidase assays with cells harbouring a plasmid-encoded P_*rtcBA*_-*lacZ* reporter and RT-qPCR. In parallel, the growth of the Hpx- stain was monitored. (A) The *rtcBA* promoter activity is induced in LB broth, LB broth supplemented with extra 5 g/L NaCl, LB broth supplemented with 50 μM Fe-citrate and nutrient broth, after 24 h under aerobic but not anaerobic conditions (*N* = 5). (B) The *rtcBA* promoter activity is induced in the Hpx- strain because of gene deletion Δ*ahpC*; RtcR, RtcB and RtcA are required for *rtcBA* promoter activity in the gene deletion mutants Δ*ahpC* (*N* = 4). (C and D) The *rtcBA* promoter activity is induced and (D) the *rtc* mRNA levels are increased, as shown by β-galactosidase reporter assays and RT-qPCR, respectively (*N* = 3). (E) The presence of the P_*rtcBA*_-*lacZ* reporter plasmid leads to inhibited growth of the Hpx- compared to the wild-type (*N* = 3). (F) In the absence of the P_*rtcBA*_-*lacZ* reporter plasmid, the inhibitory growth effect is not as evident (*N* = 3). (G) Proposed model of the molecular events occurring in the Hpx- cells in the absence (left) and presence (right) of the P_*rtcBA*_-*lacZ* reporter plasmid: absence does not interfere expression of the chromosomal Rtc genes (left), leading to normal cell growth; presence interferes with expression of the chromosomal Rtc genes (right), leading to impaired cell growth. In panels (A, B, and D), β-galactosidase activity or mRNA levels of the wild-type strain is set as 100%. Data are shown as mean and error bars represent standard deviation from the mean. *N* represents total number of independent biological replicates, with 3 technical replicates each. ANOVA ∗∗∗∗ p-value < 0.0001.

In Hpx- we observed the strongest induction of the *rtcBA* promoter to date as compared to the wild-type. This was confirmed using the P_*rtcBA*_-*lacZ* reporter plasmid and β-galactosidase assays as well as when measuring *rtcBA* mRNA levels directly in absence of P_*rtcBA*_-*lacZ* (Figures 4A and 4D). The induction was evident in all growth media under aerobic conditions, but not under microaerobic conditions, consistent with the aerobic formation of hydrogen peroxide during respiration (Figure 4A). Notably, *rtcBA* mRNA levels were not increased in presence of P_*rtcBA*_-*lacZ* (Figure 4D). We infer that this is because of multi-copy inhibition where RtcR is titrated away from the sole native chromosomal *rtcBA* promoter through binding to multiple copies of P_*rtcBA*_-*lacZ* introduced into the cells via the reporter plasmid. Multi-copy inhibition has been reported for other bacterial enhancer binding proteins, for example the NifA transcriptional activator.^28^ Subsequently, Hpx- strains with and without P_*rtcBA*_-*lacZ* were designated as Hpx-[RtcOFF] and Hpx-[RtcON], respectively (Figure 4D). In line with a prominent growth-determining role for Rtc under oxidative stress, normal growth of Hpx- was only observed when expression of chromosomal *rtcBA* was increased (compare Hpx-[RtcON] and Hpx-[RtcOFF] in Figures 4E–4G). To confirm that these observations are specific to the presence of P_*rtcBA*_-*lacZ*, we next measured the effect of another reporter plasmid, P_*hrpL*_-*lacZ.*^29^ The *hrpL* promoter is σ^54^-dependent (similar to P_*rtcBA*_) but not linked to Rtc and not found in *E*. *coli* bacteria. Indeed, in contrast to P_*rtcBA*_-*lacZ*, we neither observed activation of the *hrpL* promoter (Figure S6A), nor a Hpx- specific growth defect in presence of P_*hrpL*_-*lacZ* (Figure S6B).

We next determined whether the increased *rtcBA* promoter activity in Hpx- is attributed to the loss of a single peroxidase gene or a combination of them. We found that the deletion of *ahpC*,^30^ but not of *ahpF*, *katE* or *katG*, leads to the strong Rtc induction seen in the Hpx- background (Figure 4B). Moreover, and in line with our proposal of a regulatory RtcRAB complex, induction of *rtcBA* expression in the *ahpC* mutant is dependent on all three *rtc* genes (Figure 4B). Notably, the induction of *rtcBA* expression seems specific and closely linked to the action of glutathione reductase Gor and peroxidase AhpC rather than being part of a general response to oxidative stress given that we found no effect on *rtcBA* expression or growth in cells lacking master regulators of oxidative stress *fur*, *oxyR*, and *iscR* (Figures S6C and S6D and Table S5).

### The RtcR CARF domain is not a direct target of oxidative stress

Oxidative damage can occur to many cellular components, and so we next investigated whether the CARF domain of RtcR itself is affected by oxidative stress. We reasoned that this might lead to loss of CARF domain function and hence de-repression of the transcriptional activation by RtcR. Cysteine residues are most susceptible to oxidation, hence we assessed the presence, conservation and significance of cysteines in the CARF domain of RtcR *in silico* and *in vivo*. The *E*. *coli* RtcR CARF domain contains four cysteine residues, Cys32, Cys34, Cys91 and Cys122. Homologous CARF domains were identified using Pfam, within the family designated as RtcR (PF06956). In total, 314 bacterial CARF domain sequences generated by searching the sequence database of reference proteomes with the family hidden Markov model were assessed. The majority of these sequences contained one (35%) or two (44%) cysteines (Figure S6E), with only 17 sequences (5%) having four cysteine residues, including the *E*. *coli* RtcR CARF domain. Cysteine residues Cys34 and Cys122, but not Cys32 and Cys91, were conserved (Figure S6F). A structural model of the RtcR CARF domain was constructed using i-Tasser and visualized by Chimera (Figure S6G); however, COPA prediction of oxidation-susceptible sites indicated that none of these cysteine residues were likely susceptible to redox-mediated regulation. In line with this, cysteine to alanine substitutions, either individually or in combination, within full-length RtcR did not result in a loss of the CARF-mediated repression of transcriptional activation under non-inducing conditions (Figure S6H). In addition, the simultaneous replacement of all cysteine residues with alanine did not prevent transcriptional activation by RtcR under inducing conditions (Figure S6I). Similar to wild-type RtcR, the quadruple mutant had approximately 2-fold higher activity under inducing oxidative stress conditions as compared to non-inducing conditions, indicating that the cysteine residues are not used to sense the stress signal. Mutations C32A and C122A were predicted to cause structural perturbations according to Missense3D analysis (classified as ‘buried / exposed switch’ and ‘buried H-bond breakage’, respectively) and all mutations were predicted to decrease protein stability as assessed by Duet; however, structure comparison of the mutated with wild-type CARF domains revealed an RMSD ≤0.01 in all cases (Table S6). Therefore, both the site-directed mutagenesis and the bioinformatics analysis suggest that the cysteine residues have little effect on the CARF domain and are not involved in its inhibitory function.

Overall, our results indicate that the RtcR CARF domain senses a molecular signal generated (indirectly or directly) by oxidative stress rather than being a direct chemical target of oxidative stress. In addition, the Hpx- strain provides us with a *background leading to high Rtc induction*, *which can be utilized to further investigate the physiological signal for RtcR-dependent transcription activation*, *the cell conditions when the Rtc system is required and so it can help identify targets of the RtcB ligase*.

### The physiological signal for RtcR-dependent transcription activation

**Figure 5:**
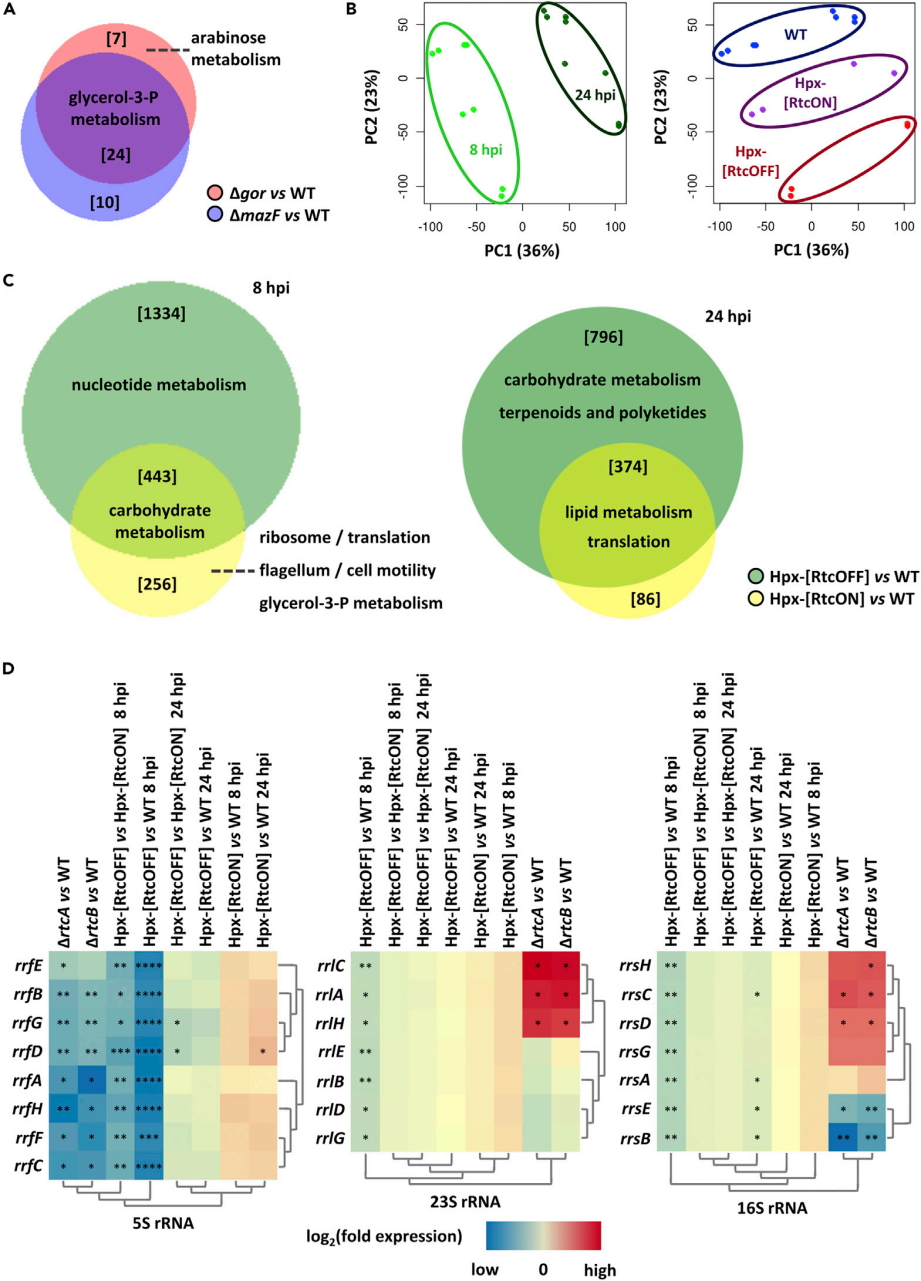
Transcriptome profiling of *E*. *coli* cells under Rtc-inducing conditions Transcriptome profiling of *E*. *coli* wild-type cells, cells lacking *gor*, *mazF* or *srmB*, and Hpx-[RtcON] and Hpx-[RtcOFF] cells was performed by NGS and followed by differential gene expression analysis. Transcriptome profiling of *E*. *coli* cells lacking *rtcA* or *rtcB* was described previously by Engl et al. (2016).^15^ (A) Venn diagram of the genes differentially expressed in cells lacking *gor* or *mazF* compared to the wild-type and associated GO terms (Tables S7 and S8). (B) Principal component analysis of the transcriptional profiling of the wild-type, Hpx-[RtcON] and Hpx-[RtcOFF] strains. (C) Venn diagram of the genes differentially expressed in Hpx-[RtcON[ and Hpx-[RtcOFF] as compared to wild-type cells at 8 and 24 hpi, and associated GO terms and KEGG pathways. (D) Heat maps of 5S (left; log_2_fold expression ±5), 23S (left; log_2_fold expression ±7) and 16S (left; log_2_fold expression ±6) ribosomal (r) RNA abundance levels in a range of mutants, as shown by NGS; rows and columns have been grouped based on a hierarchical clustering algorithm. Limma/DESeq2 ∗ adjusted p-value < 0.05; ∗∗ adjusted p-value < 0.01; ∗∗∗∗ adjusted p-value < 0.0001.

To better understand the cell states associated with *rtcBA* induction, and to help identify pathways or genes that might generate potential physiological signaling molecules that support RtcR-dependent transcription activation via the CARF domain, we used *RNA sequencing* to investigate the *global gene expression profile of selected mutants that induce the Rtc system via RtcR* (Figures 5 and S7–S10, and Tables S7–S12 and Text S3). We found that a common set of genes was differentially expressed in *E*. *coli cells lacking gor* or *mazF* (Figure 5A), indicating that similar pathways are affected (Tables S7–S8). The *srmB* mutant showed differential expression of a substantially smaller set of genes that were also distinct from those found in Δ*gor* and Δ*mazF* (Text S3 and Dataset S1). When comparing the transcriptome of Hpx-[RtcON], Hpx-[RtcOFF] and wild-type cells (Figure 5B), a differential effect on ribosome constituents was observed: the abundance of transcripts encoding ribosomal proteins was significantly decreased in Hpx-[RtcON] (Figures 5C, S9A, and S9B and Tables S9–S11), whereas no changes were detected in the levels of ribosomal RNA (Figures 5D and S7C). Conversely, the abundance of ribosomal RNAs was significantly decreased and nucleotide metabolism was affected in Hpx-[RtcOFF] cells (Figures 5D and S7C and Table S9), whereas the abundance of transcripts encoding ribosomal proteins was significantly increased (Figures S9A and S9B) as compared to both Hpx-[RtcON] and wild-type. The expression patterns of ribosomal genes observed in Hpx-[RtcOFF] were similar to those in Δ*rtcA* and Δ*rtcB* cells (Figures 5D and S7C), indicating that lack of RtcAB leads to defective ribosomes and confirming the association between Rtc and the ribosome.^15^^,^^16^ Strikingly, transcriptome analysis indicates that there are often common groups of genes whose expression levels are concomitantly affected in *E*. *coli* strains that induce *rtcBA*, and these appear to link the Rtc system to both the translation apparatus and cell wall metabolism (glyceraldehyde-3-P synthesis), even though all of the exact regulatory pathways linking these gene sets are not known. We considered that the activating ligand for RtcR is generated by known *E*. *coli* nucleotide cyclases (such as adenylate, diguanylate and GTP 3′,8′-cyclases), but we found no link between *rtcAB* inducing conditions and increased abundance of nucleotide cyclase transcripts (Figure S10).

To gain insight into potential regulatory pathways and feedback loops that might impact on RtcAB production, we examined cells lacking *rtc*^15^ for the expression of all genes whose deletion or overproduction modulates the Rtc system (Figure S11). In total, 10 out 17 of these genes were found to have significantly altered abundance in Δ*rtcA* and Δ*rtcB* and this number was significantly higher than that expected by chance, suggesting that there may be several feedback loops in place to modulate Rtc expression, with some acting through RtcR (Text S3).

### Rtc-inducing conditions correlate with loss of tRNA integrity

**Figure 6:**
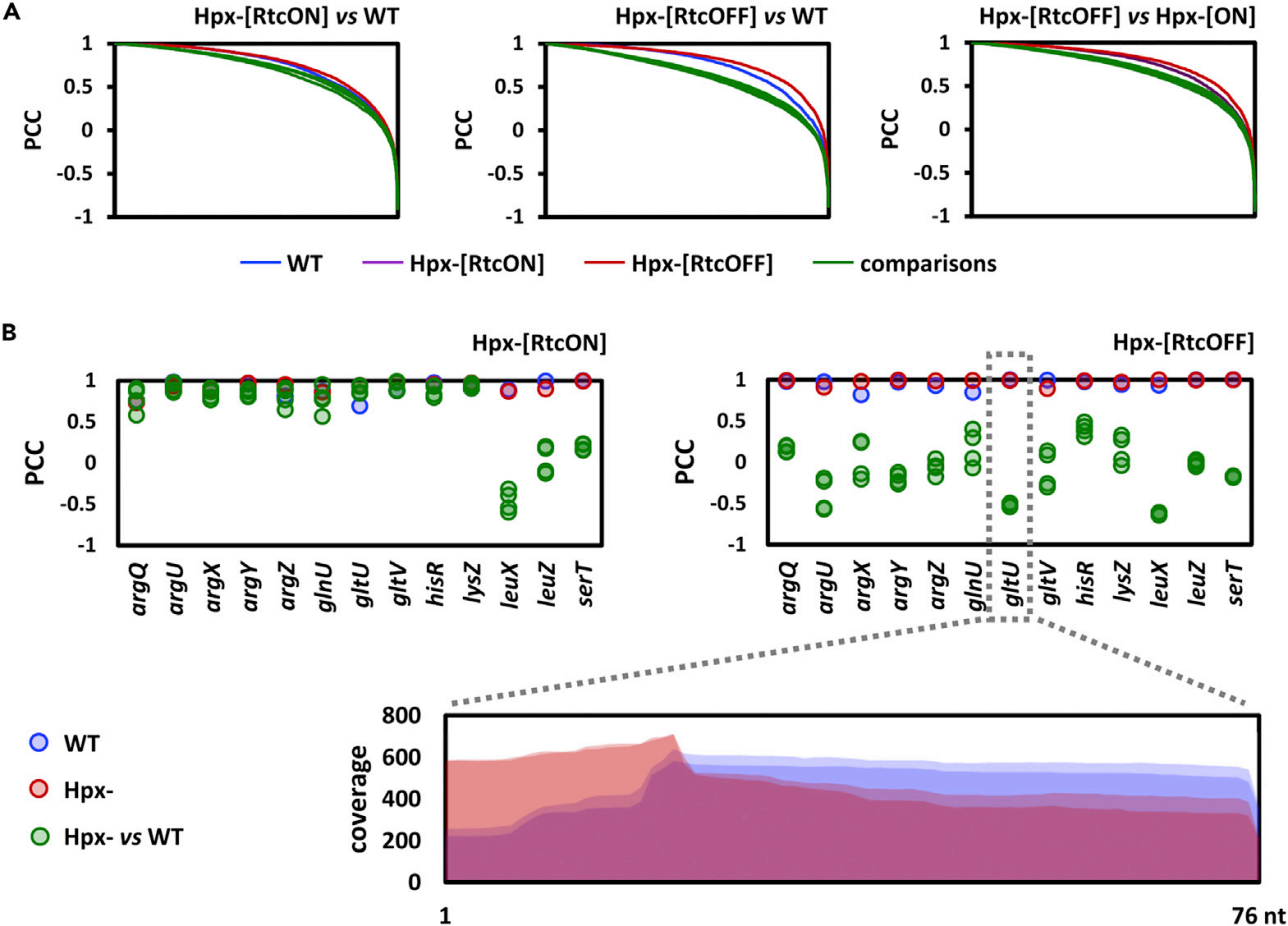
tRNAs are cleaved under oxidative stress Damaged RNAs in transcriptomes of *E*. *coli* Hpx-[RtcON] and Hpx-[RtcOFF] cells were identified by pairwise calculation of the Pearson’s Correlation Coefficient (PCC) for each individual gene. (A) Globally, more transcripts are damaged in Hpx-[RtcOFF] than Hpx-[RtcON] cells compared to wild-type cells 8 h post-inoculation, as illustrated by plotting PCC values of all genes in descending order from high to low. (B) More tRNAs are damaged in Hpx-[RtcOFF] (top right) than Hpx-[RtcON] (top left) compared to wild-type cells, as illustrated by plotting PCC values of pairwise comparisons for selected tRNAs. Distribution of all reads mapped to *gltU* in Hpx-[RtcOFF] and wild-type cells reveals no obvious cleavage sites (bottom).

Given the RNA repair capability of RtcAB and the absence of a clear cellular target to date, we searched for cleaved RNAs within the transcriptomes (Text S4). Loss of RNA integrity was evident when comparing Hpx-[RtcOFF] to wild-type at exponential (Figure 6A) and stationary phase (Figure S12D). Comparisons of Hpx-[RtcON] and Hpx-[RtcOFF] to wild-type, as well as Hpx-[RtcOFF] to Hpx-[RtcON], revealed damaged transcripts which appear to be repaired by the Rtc system. In total, 55 transcripts were identified, when comparing Hpx-[RtcOFF] to Hpx-[RtcON], including protein-coding, tRNA-coding and ncRNA-coding genes together with pseudogenes, but not rRNA-encoding genes (Figure S12C and Dataset S2). The tRNAs (Figure 6B) were the only group statistically overrepresented (p-value < 0.05), suggesting that Rtc-inducing conditions correlate with loss of tRNA integrity. Therefore, it is likely that tRNA hydrolysing events resulting from a range of stresses take place during Rtc-inducing conditions. However, there was no obvious specific tRNA cleavage site (Figure 6B) apart from the anticodon stem-loop within tRNA^fMet^ cleaved by the ribotoxin VapC^15^^,^^31^ and used for validation purposes (Figure S12A). This suggests that the tRNA damage caused by oxidative stress is not strongly localized. Damaged RNAs were also identified when comparing cells lacking *gor*, *mazF* or *srmB* to wild-type (Figure S12B). All such damaged RNAs are in principle sources of molecules containing 3′-P or 2′,3′-cyclic P termini, and so may produce CARF domain binding ligands relevant to the activation of RtcR.

### The tmRNA *ssrA* is a target of the RtcB ligase

**Figure 7:**
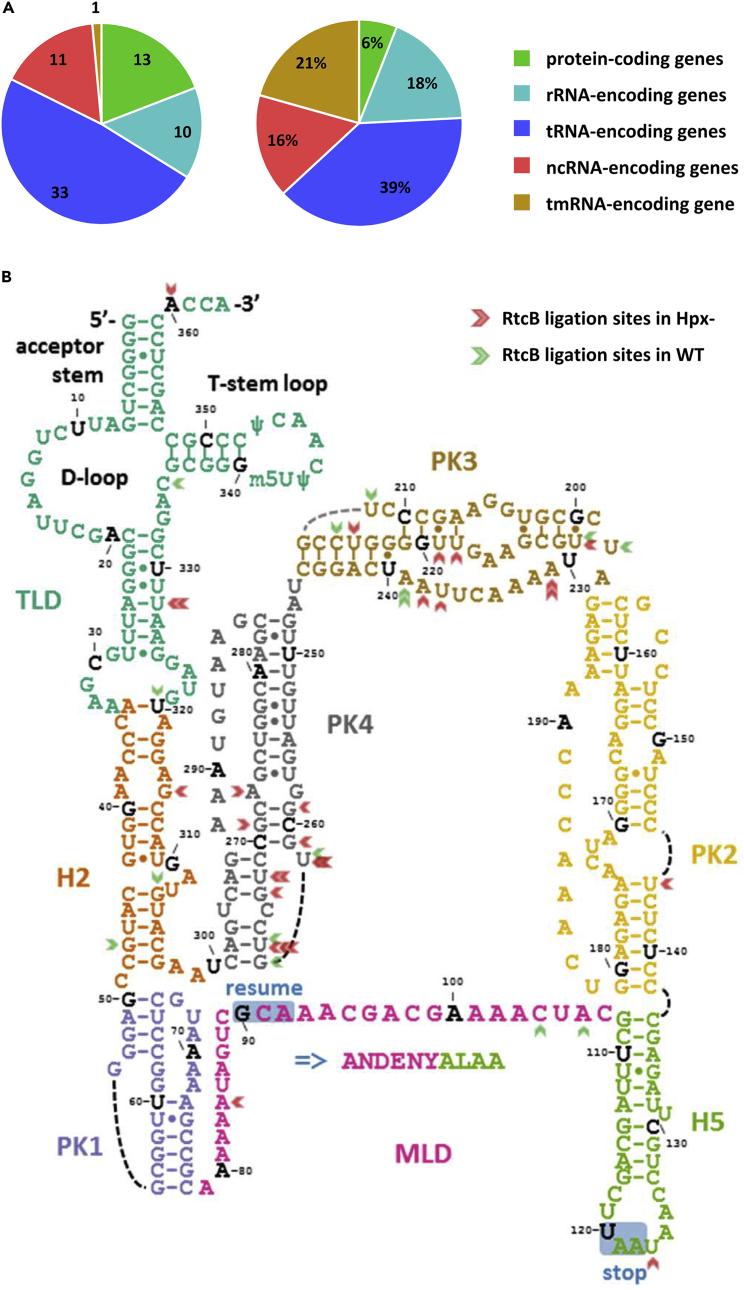
RtcB-mediated repair targets the tmRNA *ssrA* under oxidative stress Targets of the RtcB ligase, including the tmRNA *ssrA*, were identified by RtcB mediated ligation using an adapter and followed by NGS. (A) Number of genes whose RNA is an RtcB ligase target (left) and events of RtcB-mediated ligations (right). (B) Ligation events for *ssrA* in wild-type (green arrows) and Hpx- cells (red arrows). Different colours indicate distinct *ssrA* structural domains, including the tRNA-like domain (TLD), the mRNA-like domain (MLD), helices 2 (H2) and 5 (H5), and pseudoknots 1 (PK1), 2 (PK2), 3 (PK3) and 4 (PK4). The nucleotides of the open reading frame (ORF) are shown in a larger font, whereas the resume and stop codons are highlighted. Ligation events were significantly more abundant in PK3 (p-value < 0.01) for wild-type cells, and in both PK3 (p-value < 0.01) and PK4 (p-value < 0.0001) for Hpx- cells.

To directly identify RNA targets of the RtcB ligase with 3′-P or 2′,3′-cyclic P termini, we performed *in vitro* RtcB-mediated ligation reactions and RNA sequencing on purified total RNA from the Hpx- and wild-type strain. The adapter sequence was then detected in the reads, allowing us to pinpoint where RtcB-mediated ligation had taken place (Figure S13A). Sixty-eight unique RNAs were found to be RtcB targets in at least one of the eight samples investigated, with half of them identified more than once in a total of 252 ligation events (Dataset S2). Outcomes with the total RNA ligations reflect the paucity of ligatable termini in cellular RNA and not the efficiency of the ligation reaction since, when using an *in vitro* constructed RNA with a 3′-P terminus, 70% of the ends were successfully ligated. No sequence specificity was identified in either the 5′ terminus of the adaptor or the 3′ terminus of the target RNA (Figure S13B). More than 80% of the RtcB ligation targets are non-protein coding RNAs (Figure 7A), whereas only 6% of the termini correspond to protein-coding RNAs (Figure 7A).

The most frequently identified RtcB target was *ssrA* (Figure 7A), a transfer-messenger (tm) RNA that helps the ribosome overcome translation problems, such as broken mRNA or absence of the appropriate tRNA.^32^ There was a statistically significant abundance of ligation events in pseudoknots (PK) 3 and 4, as compared to the rest of the *ssrA* domains (Figure 7B); the former are particularly exposed when the tmRNA is in complex with the 30S ribosomal subunit.^33^ There was also a statistically significant increase of the ligation events in Hpx- when compared to the wild-type *E*. *coli* (Figure S13C). Notably, *ssrA* was the only small RNA whose abundance significantly increased in *E*. *coli* cells lacking *rtcB* or *rtcA* as compared to the wild-type, as illustrated by NGS and confirmed by RT-qPCR amplification (Figures S13D and S13E). If the Rtc system plays a role in tmRNA maintenance, then lack of RtcA/RtcB enzymatic activities might well stimulate tmRNA expression to compensate for the damaged molecules that cannot be repaired. No significant changes were observed in *E*. *coli* cells lacking *gor*, *mazF* or *srmB*, where the Rtc system is induced (Table 1); in contrast, the abundance of *ssrA* was significantly reduced in Hpx-[RtcOFF] compared to Hpx-[RtcON] cells 8 h post-inoculation (Figure S13E). Taken together, we conclude that *in vivo* RtcB only acts on a small number of mostly non-protein-coding RNAs, in particular on *ssrA*, the tmRNA involved in the rescue of stalled ribosomes.

### RtcB increases translation elongation speed but has no effect on translation fidelity

**Figure 8:**
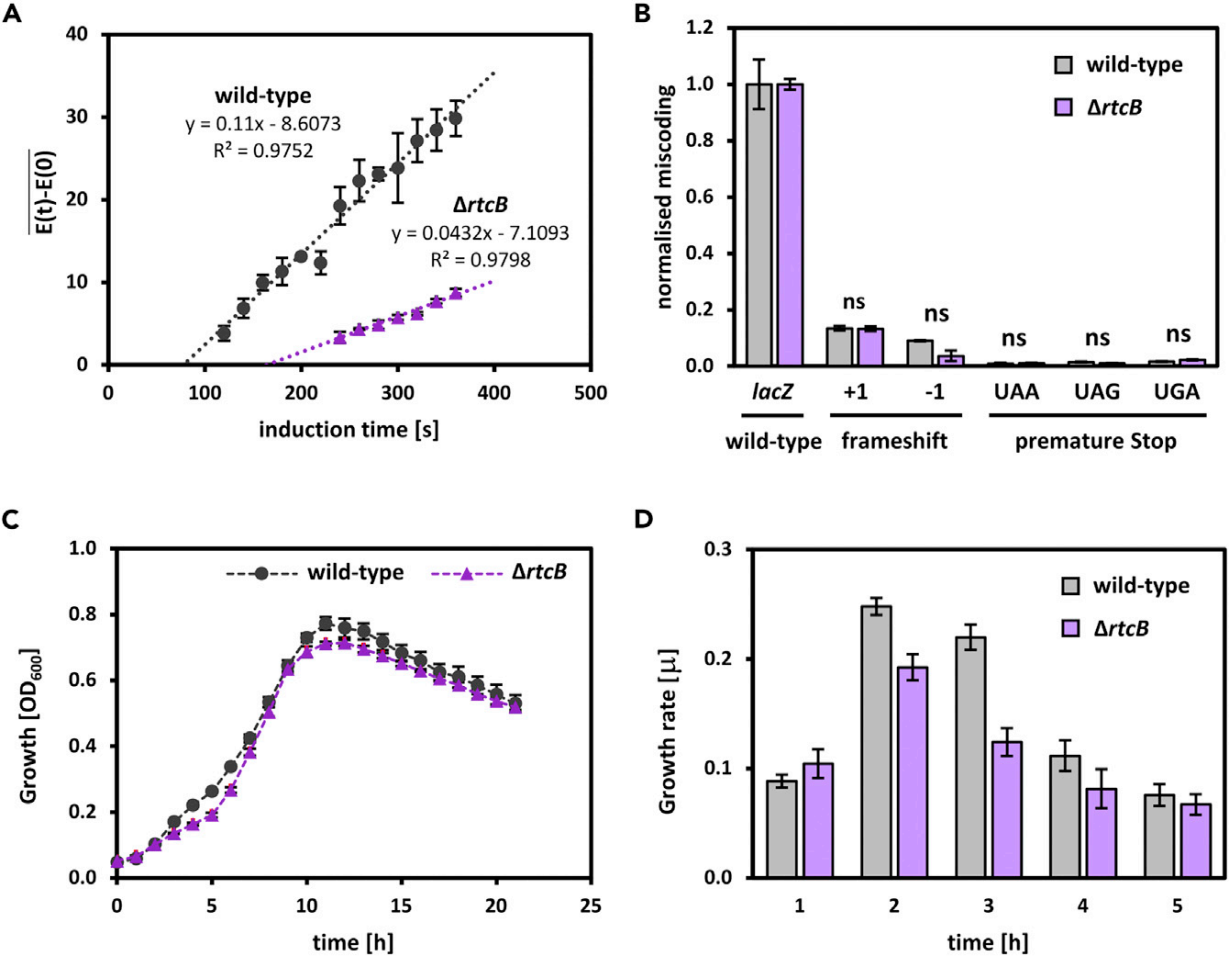
RtcB increases translation elongation speed *in vivo* The impact of RtcB on the functioning of the translation apparatus was measured *in vivo* using β-galactosidase based assays and growth assays. RtcB (A) increases translation elongation speed, (B) has no effect on translation fidelity, (C and D) increases cell growth and (D) increases cell growth rate in early exponential phase. Data are shown as mean and error bars represent standard deviation from the mean of triplicates. ns = non-significant (p-value > 0.05).

Given that *ssrA* is a target of RtcB ligation (Figures 7 and S14), and that *rtcBA* promoter activity is repressed by the ribosome-protecting ABC-F proteins YheS^34^ and its paralogs EttA (formerly YjjK), Uup, YbiT and (Figures S14A–S14C), we tested whether RtcB impacts ribosome function. Indeed, the speed of translation elongation was reduced by approximately 50% in cells lacking *rtcB* (4.7 aa/s) compared to wild-type (9.9 aa/s) (Figure 8A). However, translation was equally accurate in both strains (Figure 8B). Thus RtcB acts rather specifically to maintain translation elongation speed but appears not to be important for the fidelity of translation and we infer that this effect on translation is related to the role of RtcB in maintaining tRNA and *ssrA* integrity as described above. In line with the decreased translation elongation speed, cells lacking *rtcB* had a markedly reduced growth rate under the conditions when the RtcB’s impact on translation was measured (growth in minimal medium and early exponential phase, see Figure 8D). At later stages, growth of the *rtcB* mutant picks up again (Figure 8C), suggesting either that RtcB action is particularly important at early exponential growth or that the cells manage to compensate for the loss of *rtcB*. The latter scenario might be explained by the presence of multiple fail-safe systems that *E*. *coli* cells employ to rescue impaired ribosomes.^35^ We also note that EttA (formerly YijK), one of the ABC-F proteins which represses *rtcBA* expression, gates the entry of ribosomes into the elongation cycle^36^ indicating synergies between the need for RtcB and the actions of EttA in maintaining translation elongation.

## Discussion

**Figure 9:**
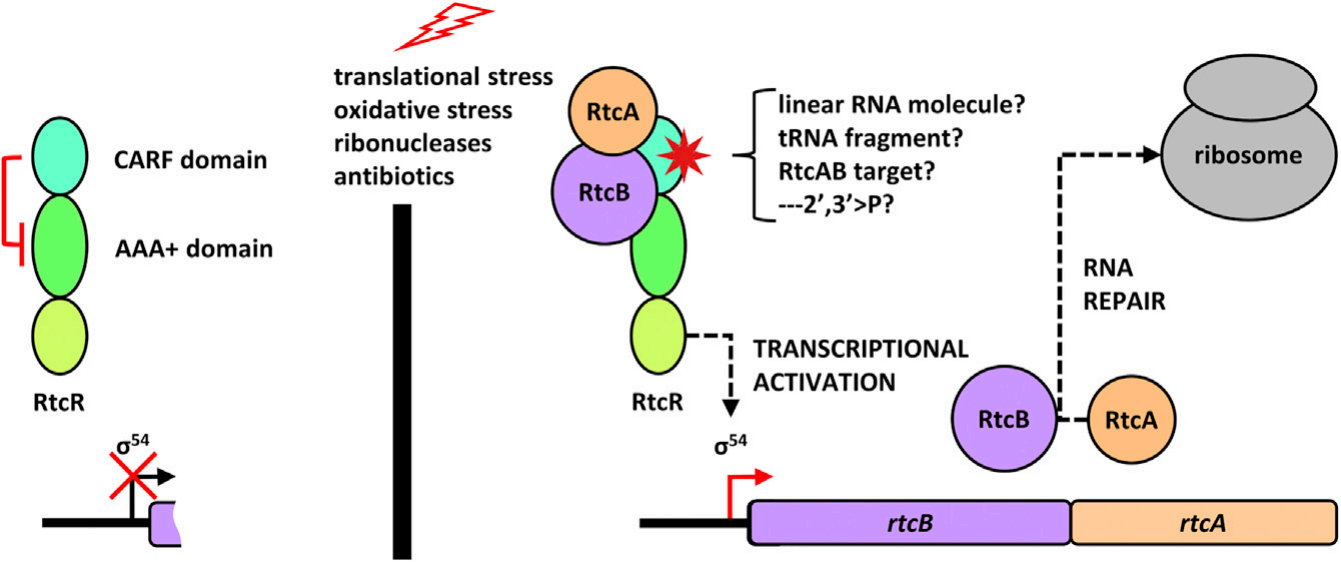
Regulation of Rtc RNA repair system expression and function Under conditions that activate of the Rtc RNA repair system that helps maintain translation, RtcA and RtcB interact with the RtcR CARF domain together with an unknown ligand, leading to derepression of the RtcR AAA + domain by the RtcR CARF domain. We propose that the complex has dual functionality: transcriptional activation of *rtcBA* by RtcR and RNA repair by RtcA and RtcB. The RtcR CARF domain likely does not use cOAs as activating ligands (this work), rather it binds particular linear RNA sequences with a 2’-3’ cyclic P end (this work and Hughes et al., 2020^18^).

In this study we provide novel insights into how bacteria mount RtcBA-dependent RNA repair in response to factors that compromise RNA integrity such as oxidative stress (Figure 9).

Dealing with oxidative stress is crucial to bacterial pathogenicity where host defenses attack invading bacteria, as well as in aerobic culturing of bacteria in many environmental and technological settings. RNA, similar to DNA and proteins, is attacked by reactive oxygen species; however, unlike DNA and proteins, the impact of oxidative stress on RNA is understudied. To date, oxidative stress damage to RNA has been mostly linked to nitrogenous base modifications rather than breakage of the phosphodiester bonds, although tRNA and rRNA cleavage has been reported in eukaryotes^37^^,^^38^^,^^39^ and bacteria.^40^ In this work we identified the tmRNA *ssrA* as an RtcB substrate during oxidative stress suggesting that the Rtc system plays a role in the quality control of the translation apparatus under these conditions. At this stage it is not clear if tmRNA damage under oxidative stress is a result of direct oxidation or of activation of a nuclease that uses tmRNA as a substrate. The tmRNA precursor molecule is known to be cleaved at the 5′ and 3’ terminus by RNase P and RNase E, respectively, resulting in the mature tmRNA under study.^41^^,^^42^ The tmRNA *ssrA* is also cleaved in a ribosome-dependent manner by the RelE toxin,^43^ whose abundance was significantly increased in Hpx- cells at 8 h postinoculation. RelE does not appear to have any sequence preference *in vivo*,^44^ in line with the diffuse set of RtcB dependent ligation sites across the tmRNA molecule.

Bacteria experience oxidative stress^45^^,^^46^ including oxidative damage to RNA^47^ also in the presence of antibiotics. Previously, we showed that *E*. *coli* cells lacking one of the *rtc* genes are more sensitive to several antibiotics that target ribosomes and the cell wall15. This suggests that the Rtc system constitutes a functional link between the translation apparatus and the cell envelope. Our current work on oxidative stress may now provide the missing link between Rtc induction and antibiotic-targeting of cell wall synthesis, should these agents themselves lead to oxidative stress. This link may be direct, if the antibiotics themselves generate an oxidant, or somewhat indirect, if the translation apparatus becomes sensitive to oxidants.

How does Rtc then sense the RNA damage caused by oxidative stress? Our results show that signaling via the CARF domain of RtcR deviates from other CARF domain containing proteins that e.g. modulate CRISPR-Cas systems.^4^^,^^5^^,^^48^^,^^49^ Fragments of damaged tRNAs with a cyclic phosphate 5’ end can bind with low affinity to RtcR *in vitro* and are proposed to alleviate the CARF-mediated repression of RtcR.^18^ We have now demonstrated that RtcA and RtcB also directly bind to RtcR via its CARF domain and help activate *rtcBA* expression. Interactions between RtcR and RtcAB may also facilitate their access to damaged RNA ends, possibly increasing affinity for such RNAs. The conditional requirement for RtcA to stimulate expression could reflect the type of RNA ends generated under Rtc inducing conditions; RtcB is capable of ligating 3′-P and 2′,3′-cyclic P ends but not 2’-P ends that should be previously modified by RtcA. To date, the full extent of CARF domain signaling systems is unknown.^4^^,^^5^^,^^50^ Yet, the *in trans* interactions of the CARF domain of RtcR with partner proteins as well as particular RNA ligands now suggest that CARF domain containing proteins can be part of novel signaling networks that do not necessarily rely on Palm domain containing proteins to generate cOAs as activating ligands, as observed for CRISPR-Cas systems. In *E*. *coli*, Palm domains are present in diguanylate cyclases, but in our studies there was no obvious link between Rtc inducing conditions and expression of these genes (Figure S10). Our findings now open the possibility that CARF domains within other systems in RNA biology are regulated through an *in trans* acting partner protein and not solely through the binding of particular linear or cyclic RNA ligands. Furthermore, a reciprocal control of the partner proteins may be achieved by the CARF domain, leading to a subtle control of overall enzymatic outputs of the partner proteins, in this case the activities of RtcAB as modulated by RtcR.

### Limitations of the study

The range of Rtc inducing conditions explored here will be united through the formation of one or perhaps a small set of ligands that act directly on transcriptional activator RtcR to stimulate its activity. The precise pathways and mechanisms that result in the formation of such activating ligands and those that can then degrade such signals remain largely unknown, and so the points at which various Rtc inducing stresses act is still speculative. A full biochemical recapitulation of a Rtc inducing event remains to be established, to help define the precise mechanism operating for signal generation and propagation as well as the atomic structure of activating ligands. Such biochemical recapitulation with purified components would be necessary to fully understand the roles of the RtcR to RtcA and RtcB interactions described in this report.

## STAR★Methods

### Key resources table

**Table:** 

REAGENT or RESOURCE	SOURCE	IDENTIFIER
**Antibodies**		

monoclonal mouse anti-polyhis-peroxidase antibody	Sigma	A7058; RRID: AB_258326
monoclonal mouse anti-his antibody	Bio-Rad	AD1.1.10
monoclonal mouse anti-MBP probe antibody	Santa Cruz Biotechnology	sc-13564; RRID: AB_675707
sheep anti-mouse antibody-horseradish peroxidase	Merck	NXA931V

**Bacterial and virus strains**		

*Escherichia coli* K-12 MG1655	Yale Culture Collection	N/A
*Escherichia coli* K-12 MG1655 derivatives	See Table S2	N/A
*Escherichia coli* K-12 BW25113	Datsenko et al., 2000^61^	N/A
*Escherichia coli* K-12 BW25113 derivatives	Baba et al., 2006^52^	N/A
*Escherichia coli* BTH101	BACTH System Kit	N/A

**Chemicals, peptides, and recombinant proteins**		

RtcB ligase	New England Biolabs	M0458S
RQ1 RNase-free DNase	Promega	M6101
SuperScript III Reverse Transcriptase	ThermoFisher Scientific	18080044
Power SYBR Green PCR Master Mix	Applied Biosystems	4367660
Pierce Premium Grade dithiobis(succinimidyl propionate)	ThermoFisher Scientific	PG82081
native PAGE sample buffer	ThermoFisher Scientific	BN2003

**Critical commercial assays**		

Bacterial Adenylate Cyclase Two-Hybrid System Kit	Euromedex	EUK001
RNeasy Protect Bacteria mini kit	Qiagen	76506/74104
Monolith Protein Labelling Kit RED-NHS 2nd Generation (Amine Reactive)	NanoTemper Technologies	MO-L011
Westar Supernova HRP Detection Substrate	Cyanagen	K1-0068

**Deposited data**		

*Escherichia coli* K-12 MG1655 genome	NCBI Reference Sequence	NC_000913
Transcriptome profile of *Escherichia coli* K-12 MG1655	This study	GSE165118

**Oligonucleotides**		

qPCR primers for *rtcB* F: 5’-ACGTGATAAAGGTGCCTGGG-3’ R: 5’-CACACCTGGTCCGACTCATC-3’	Engl et al., 2016^15^	N/A
qPCR primers for *rtcA* F: 5’-GACCAACTGGTGCTACCGAT-3’ R: 5’-GCGTTACGCCATCTGTTTCT-3’	Engl et al., 2016^15^	N/A
qPCR primers for *rtcR* F: 5’-GGTCATCGATCGACTGGAAT-3’ R: 5’-TCAATCTCAACGCTCACCAC-3’	This study	N/A
qPCR primers for *ssrA* F: 5’-AGTCGCAAACGACGAAAACT-3’ R: 5’-GCGATCTCTTTTGGGTTTGA-3’	This study	N/A
adapter for RtcB-mediated ligation 5’-NNNNTGGAATTGTCGGGTGCCAAGG-3’	This study	N/A
tRNA^Glu[UUC]^ 35-mer 5’-ACUCCGAUAUCACGCUUUCACCGUG AUAUCGGAGU-3’	This study	N/A
tRNA^Glu[UUC]^ 17-mer 5’-ACUCCGAUAUCACGCUU-3’	This study	N/A

**Recombinant DNA**		

Plasmid vectors and derivatives	See Table S1	N/A

**Software and algorithms**		

FIJI/ImageJ	Schindelin et al., 2012^79^	https://imagej.net/software/fiji/
progressiveMauve	Darling et al., 2010^64^	https://darlinglab.org/mauve/user-guide/progressivemauve.html
FASTQC	Babraham Bioinformatics	https://www.bioinformatics.babraham.ac.uk/projects/fastqc/
Trimmomatic	Bolger et al., 2014^65^	http://www.usadellab.org/cms/?page=trimmomatic
Bowtie 2	Langmead and Salzberg, 2012^66^	http://bowtie-bio.sourceforge.net/bowtie2/index.shtml
HTSeq	Anders et al., 2015^67^	https://htseq.readthedocs.io/en/master/
DESeq2 R/Bioconductor package	Love et al., 2014^68^	https://bioconductor.org/packages/release/bioc/html/DESeq2.html
FASTX toolkit	Hannon Lab	http://hannonlab.cshl.edu/fastx_toolkit/
SAM tools	Li et al., 2009^69^	http://www.htslib.org/
Integrative Genomics Viewer (IGV)	Thorvaldsdóttir et al., 2013^70^	https://software.broadinstitute.org/software/igv/
PANTHER Classification System for GO	Mi et al., 2013^71^	http://geneontology.org/
KEGG Automatic Annotation Server (KAAS)	Moriya et al., 2007^73^	http://www.genome.jp/kegg/kaas/
BioVenn	Hulsen et al., 2008^74^	https://www.biovenn.nl/
Weblogo	Crooks et al., 2004^75^	https://weblogo.berkeley.edu/logo.cgi
Iterative Threading ASSEmbly Refinement (I-TASSER)	Yang et al., 2015^76^	https://zhanggroup.org/I-TASSER/
UCSF Chimera	Pettersen et al., 2004^77^	https://www.cgl.ucsf.edu/chimera/
Cysteine Oxidation Prediction Algorithm (COPA)	Sanchez et al., 2008^78^	N/A
Missense3D	Ittisoponpisan et al., 2019^79^	http://missense3d.bc.ic.ac.uk/
DUET	Pires et al., 2014^80^	http://structure.bioc.cam.ac.uk/duet
GraphPad Prism 6	Dotmatics	https://www.graphpad.com/dl/96314/10B92408/

### Resource availability

#### Lead contact

Further information and requests for resources and reagents should be directed to and will be fulfilled by the lead contact, Dr Ioly Kotta-Loizou (i.kotta-loizou13@imperial.ac.uk).

#### Materials availability

All plasmids generated and/or used in this study are listed in Table S14. All plasmids generated in this study are available from the lead contact on request.

### Experimental model and subject details

#### Microbe strains

All bacterial strains based on *E*. *coli* K-12 MG1655, constructed and/or used in this study, are listed in Table S13. Bacteria were grown in Luria-Bertani (LB) rich medium with or without additional NaCl or Fe-citrate, nutrient (N) medium or supplemented M9 minimal medium at 37°C. Microaerobic conditions were established and verified as described previously.^51^ Antibiotics were used to a final concentration of 100 μg/mL for ampicillin, 25–50 μg/mL for kanamycin and 30 μg/mL for chloramphenicol. Expression inductions were performed using 0.02–0.2% (w/v) arabinose for the pBAD18 cm plasmids, 1 mM IPTG for the pCA25N plasmids and 0.5 mM IPTG for the pET and pMALc2 plasmids.

### Method details

#### KEIO library strain assessment

*E*. *coli* K-12 MG1655 stains carrying the in-frame gene deletions derived from the KEIO collection^52^ were constructed by generalized P1_*vir*_ phage transduction (Table S13). The P_*rtcBA*_-*lacZ* reporter plasmid was introduced into the gene deletion mutants and levels of Rtc induction were assessed using β-galactosidase assays. The chromosomal low level native *lacZ* levels were also assessed in gene deletion mutants without the P_*rtcBA*_-*lacZ* reporter plasmid to establish that the observed elevated β-galactosidase activity was Rtc specific (Table S1).

#### ASKA library screening

Screening of the ASKA library^53^ was performed by introducing the P_*rtcBA*_-*lacZ* reporter plasmid into the ASKA strains carrying the pCA25N expression plasmids with the *E*. *coli* open reading frames (ORFs); following transformation, the strains were plated on LB agar plates with X-gal for blue/white colony screening. Colonies lighter or darker in colour than the strain carrying the empty pCA25N expression plasmid were collected, Rtc induction was verified using β-galactosidase assays and the pCAN25N plasmids were sequenced to determine the ORF. The chromosomal constitutive green fluorescent protein (GFP) levels were also assessed in over-expressor strains to prove that the observed elevated β-galactosidase activity was Rtc specific (Table S2).

#### Bacterial 2-hybrid assays

Bacterial 2-hybrid assays were performed using the Bacterial Adenylate Cyclase Two-Hybrid (BACTH) System Kit (Euromedex) according to the manufacturer’s instructions. Full-length RtcB, RtcA and RtcR together with truncated forms of RtcR lacking one or two functional domains (RtcR_1-186_, RtcR_1-353_, RtcR_187-353_, RtcR_187-532_, RtcR_354-532_) were cloned in fusion with at either the N or the C-termini of the T18 fragment (vectors pUT18 and pUT18C) or of the T25 fragment (vectors pKT25 and pKNT25). The plasmids were co-transformed in pairs in the reporter strain for BACTH assay BTH101 and interactions between the hybrid proteins leading to *lacZ* reporter expression were monitored by β-galactosidase assays. Empty pUT18, pUT18C, pKT25 and pKNT25 vectors co-transformed in pairs were used as negative controls. Plasmids pKT25-zip and pUC18C-zip were used as positive controls. Plasmids pUT18C-HrpS, pUT18C-HrpV, pKT25-HrpS and pKT25-HrpV^54^ were used to confirm the specificity of the observed interactions.

#### Β-galactosidase assays

Activity of β-galactosidase in bacterial cells was measured as described previously,^55^ following growth of bacterial strains in LB or M9 medium for up to 48 h.

#### Reverse transcriptase – Quantitative polymerase chain reaction (RT-qPCR) assays

Total bacterial RNA was extracted using the Qiagen RNeasy Protect Bacteria mini kit and treated with DNase I (Promega) and reverse transcription was performed using SuperScript III Reverse Transcriptase. The RT-qPCR assays were performed in the OneStepPlus Real-Time qPCR System (Applied Biosystems) using the Power SYBR Green PCR Master Mix (Applied Biosystems). Selected genes were amplified using target-specific primer pairs as described previously.^15^

#### Ribosome profiling

Ribosome profiling was conducted under ribosome-associative conditions, as described previously.^15^

#### Protein overexpression and purification

Either pET vectors expressing N-terminal his-tagged RtcA and RtcB or a pMALc2 vector expressing RtcR N-terminally tagged with maltose binding protein (MBP; Table S2) were introduced into *E*. *coli* BL21 cells. Induction, cell sonication using the VCX 130 Ultrasonic Processor (Sonics & Materials, Inc.) and protein purification using the AKTA Fast Protein Liquid Chromatography (FPLC) system (GE Healthcare Life Sciences) were performed as described previously.^56^

#### Gel filtration

Gel-filtration chromatography (Superdex 200, 10 by 300 mm; GE Healthcare Life Sciences) was carried out as described previously.^56^ Globular proteins of known molecular weight (thyroglobulin, 669 kDa; apoferritin, 443 kDa; β-amylase, 200 kDa; alcohol dehydrogenase, 150 kDa; bovine serum albumin, 66 kDa; carbonic anhydrase, 29 kDa) were used as markers.

#### Protein crosslinking

For protein crosslinking *in vitro*, 5 μg of interacting proteins in 20 mM HEPES buffer (pH 7.5) in a total volume of 10 μL were treated with 0.5 μL of 0.25% freshly prepared solution of glutaraldehyde for 3 min at 37°C. The reaction was terminated by addition of 1 μL of 1 M Tris-HCl (pH 8.0). For protein crosslinking *in vivo*, cells were grown to exponential phase, washed twice with PBS and incubated in the presence of 100 μM Pierce Premium Grade dithiobis(succinimidyl propionate) (DSP; Thermo Fisher Scientific) for 30 min at 25°C. The reaction was terminated by addition of 20 mM Tris-HCl (pH 8.0), as described previously.^57^ DSP crosslinking was reversed using dithiothreitol (DTT) according to the manufacturer’s instructions.

#### Microscale thermophoresis (MST)

All MST experiments were performed using a Monolith NT.115 instrument (NanoTemper Technologies, Germany) at 24°C. Modified PBS (250 mM NaCl, 2.7 mM KCl, 10 mM Na_2_HPO_4_, 1.8 mM KH_2_PO_4_) supplemented with 0.05% Tween-20 was used as MST-binding buffer for all experiments. Standard treated capillaries were used. RtcA, RtcB, RtcR_WT_ and RtcR_ΔCARF_ were purified to homogeneity as described above. MBP tagged RtcR_WT_ and RtcR_ΔCARF_ were labeled with the kit Monolith Protein Labeling Kit RED-NHS 2nd Generation (Amine Reactive; NanoTemper Technologies). Labeled MBP-RtcR_WT_ or RtcR_ΔCARF_ were diluted to a concentration of 50 nM and mixed with an equal volume of a serial dilution series of RtcA or RtcB and then incubated at room temperature for 20 min before loading into MST capillaries. Single MST experiments were performed using 60% LED power and 20% MST power with a wait time of 5 s, laser on time of 30 s and a back-diffusion time of 5 s. MST data were analyzed in GraphPad Prism and the data were fitted with the Hill equation. The mean half effective concentration (EC_50_) values were calculated with standard error (SE). Each experiment was repeated at least three times.

#### SDS-PAGE and immunoblotting

Proteins were visualized on 6–12% (w/v) SDS-polyacrylamide gels electrophoresed in Tris-Glycine-SDS buffer following staining with Bio-Safe Coomassie Stain (Bio-Rad). Immunoblotting to detect His-tagged RtcA and RtcB was performed using a monoclonal mouse anti-polyhistidine-peroxidase antibody (A7058, Sigma) or a monoclonal mouse anti-histidine antibody (AD1.1.10, Bio-Rad) followed by a sheep anti-mouse antibody conjugated to horseradish peroxidase (NXA931V, Merck). Immunoblotting to detect MBP-tagged RtcR was performed using a monoclonal mouse anti-MBP probe antibody (R29.6; sc-13564, Santa Cruz Biotechnology) followed by the aforementioned secondary antibody. In all cases, peroxidase activity was detected using the chemiluminescent substrate Westar Supernova (Cyanagen) according to the manufacturer’s instructions. Image analysis was performed using the FIJI/ImageJ image processing package (Schindelin et al., 2012).^81^

#### *In vitro* RtcB ligation

Total bacterial RNA was extracted using the Qiagen RNeasy Protect Bacteria mini kit and adapter 5′-NNNNTGGAATTGTCGGGTGCCAAGG-3′ was used in RtcB-mediated ligation reactions to identify 3′-P or 2′,3′-cyclic P RNA ends that would be RtcB targets as described.^58^ An *in vitro* synthesized RNA with a 3′-P terminus was processed in parallel as a positive control. Commercially available RtcB Ligase (New England Biolabs) was used according to the manufacturer’s instructions and the reactions were subjected to RNA sequencing.

#### Small primed (sp) RNA assay for *in vitro* transcription

Reactions were performed in 10 μL final volumes containing 100 nM σ^54^ RNA polymerase holoenzyme (1:4 ratio of RNAP:σ^57^) and 20 nM of promoter DNA as supercoiled plasmid in STA buffer,^59^ and incubated at room temperature. The spRNA synthesis was initiated by adding 0.5 mM dinucleotide primer ApC or UpG (for native P_*rtcBA*_ or hybrid P_*rtcBA*_-_*nifH*_ promoters respectively) and 0.2 mCi/mL [α-^32^P] ATP or GTP (3000 Ci/mmol). The reaction mixtures were quenched by addition of 4 μL of denaturing loading buffer, electrophoresed on a 20% (w/v) urea-PAG and visualized using a Fuji FLA-5000 Phosphor imager. Image analysis was performed using the FIJI/ImageJ image processing package (Schindelin et al., 2012).^79^

#### Electrophoretic mobility shift assay (EMSA)

Fragments of tRNA^Glu[UUC]^ fluorescently labeled at the 5′ terminus with Cy3 were ordered from Sigma. The 35-mer comprises the whole anticodon stem-loop and has a 3′-OH; the 17-mer comprises half the anticodon stem-loop and has a 3′-P. The fragments (50 nM) were incubated with purified RtcR full length or RtcR CARF (up to 5 mM) in binding buffer (50 mM Na-glutamate, 50 mM NaCl, 20 mM Tris-Cl pH 8, 8 mM MgCl_2_, 0.1 mM DTT, 5% glycerol) and incubated at room temperature. The reaction mixtures were mixed with native PAGE sample buffer (Thermo Fisher Scientific) and visualized on a 4% (w/v) native PAGE using a Fuji FLA-5000 Phosphor imager.

#### Translation elongation speed

The speed of translation elongation was measured *in vivo* via LacZα complementation using a 774 amino acid-long FusA-LacZα fusion protein as reporter.^60^ The measurements were done in *E*. *coli* BW25113 wild-type and Δ*rtcB* strains.^52^^,^^61^ The Δ*lacZ4787*(::*rrnB-3*) genotype renders BW25113 LacZ-deficient enabling background-free β-galactosidase assays. The strains were grown to mid-exponential phase (OD_600_ ∼ 0.5) in M9 minimal medium supplemented with 10 μg/mL gentamicin. Expression of FusA-LacZα was induced with 5 mM IPTG and 500 μL samples were taken at 20 s time intervals. Translation was blocked immediately with 10 μL of 34 mg/mL chloramphenicol and the samples were frozen in liquid nitrogen and stored at −80°C. LacZα complementation was enabled for 1 h at 37°C prior to subjecting the samples to the fluorescent β-galactosidase substrate 4-methylumbelliferyl-D-galactopyranoside (MUG). The reaction mix contained 400 μL of sample, 100 μL of 5× Z-Buffer and 100 μL of 4 mg/mL MUG. The reaction was stopped after 2 h with 300 μL of 1 M Na_2_CO_3_ and the fluorescence intensity was measured at 365 nm excitation and 450 nm emission. The translation time of FusA-LacZα was extracted via the X-intercept of a Schleif plot (square root of newly synthesized FusA-LacZα vs induction time) and the speed of translation elongation was calculated as the ratio between the length and translation time of FusA-LacZα.

#### Translation fidelity

Translation fidelity was monitored *in vivo* in *E*. *coli* K-12 BW25113 wild-type and Δ*rtcB* strains via the production of functional β-galactosidase as described,^62^^,^^63^ using reporter plasmids with either frameshifting errors or premature stop-codons near the 5-end of *lacZ*. Strains were grown to mid-exponential phase (OD_600_ ∼ 0.5) in M9 minimal medium supplemented with 12.5 μg/mL tetracycline and β-galactosidase assays were performed as described.^62^ Translation fidelity was expressed as normalized miscoding^62^^,^^63^ using Miller units from strains harboring the wild-type *lacZ* reporter plasmid for normalization.

#### Next generation sequencing (NGS)

Genome sequencing of the *E*. *coli* Hpx- strain was performed by MicrobesNG, Birmingham, UK, including genomic DNA extraction, library preparation, Illumina sequencing, genome assembly and annotation. Alignment of the resulting contigs to the *E*. *coli* K-12 MG1655 genome (NCBI Reference Sequence: NC_000913) and single nucleotide polymorphism (SNP) analysis was performed using the progressiveMauve algorithm.^64^ RNA sequencing of the wild-type and Hpx- strains together with *E*. *coli* strains lacking the *gor*, *mazF* or *srmB* gene was performed by Vertis Biotechnologie, including total RNA extraction, ribosomal RNA depletion, library preparation and Illumina sequencing. The overall quality of the sequencing data was assessed using FASTQC and the adapters and low-quality bases were clipped and trimmed respectively from the reads using Trimmomatic.^65^ The reads were then mapped to the *E*. *coli* K-12 MG1655 genome (NCBI Reference Sequence: NC_000913) using Bowtie 2^66^ and quantified using HTSeq.^67^ Differential expression analysis was performed using the DESeq2 R/Bioconductor package.^68^ RNA sequencing of the RtcB-mediated ligation reactions was performed by Vertis Biotechnologie as previously described. Following quality control with FASTQC and Trimmomatic as described above, reads with the ligated adapter were selected and retained by a simple custom Perl script using a regular expression to identify the adapter within the read sequence. The adapter was subsequently removed using the CLIPPER function of the FASTX toolkit (http://hannonlab.cshl.edu/fastx_toolkit/), so that only the *E*. *coli* transcript sequence remained for further processing. Mapping and quantification of the clipped reads was performed as described above. SAM tools^69^ were used for the conversion and indexing of .sam/.bam files and alignments were visualized with Integrative Genomics Viewer (IGV).^70^ The number of reads that aligned to each position of the genome was calculated using the count function of IGV tools with the window size set to 1. Custom scripts were written in Perl and all functions were performed in Ubuntu.

#### Computational analyses

Gene ontology (GO) enrichment analysis was performed using the PANTHER Classification System.^71^ Kyoto Encyclopedia of Genes and Genomes (KEGG) pathway analysis^72^ was performed using the KEGG Automatic Annotation Server (KAAS).^73^ BioVenn was used to create area-proportional Venn diagrams.^74^ Weblogo was used to visualize graphical representations of amino acid or nucleic acid multiple sequence alignments.^75^ Protein structure modeling by iterative threading assembly simulations was performed using Iterative Threading ASSEmbly Refinement (I-TASSER).^76^ Molecular graphics images were produced using the UCSF Chimera package^77^ from the Computer Graphics Laboratory, University of California, San Francisco (supported by NIH P41 RR-01081). The potential of cysteine residues for disulphide bridge formation was assessed using the Cysteine Oxidation Prediction Algorithm (COPA).^78^ Structural changes introduced by amino acid substitutions were predicted using Missense3D.^79^ Changes to protein stability on mutation were predicted using DUET.^80^

### Quantification and statistical analysis

All data are shown in figures as mean and error bars represent standard deviation from the mean. Details of each experiment, including the number of replicates (*N*) and the statistical test used can be found in the corresponding figure legends. Analysis of variance (ANOVA) was performed using the GraphPad Prism 6 software. Hypergeometric tests were performed using R/Bioconductor. Differences were considered statistically significant if the p-value was less than 0.05; ∗ indicates p-value < 0.05; ∗∗ indicates p-value < 0.01; ∗∗∗ indicates p-value < 0.001; ∗∗∗∗ indicates p-value < 0.0001.

## Data Availability

• Next-generation sequencing data have been deposited at Gene Expression Omnibus (GEO) and are publicly available as of the date of publication at GEO: GSE165118 (https://www.ncbi.nlm.nih.gov/geo/query/acc.cgi?acc=GSE165118). • This article does not report original code. • Any additional information required to reanalyze the data reported in this article is available from the lead contact on request.

## Supplemental information

Document S1. Figures S1–S14, Tables S1–S14, and Data S1–S3

Dataset S1. RNA sequencing – differential expression analysis, related to Figure 5

Dataset S2. RNA sequencing – identification of damaged RNAs, related to Figure 6
