# Supplementary material for: The RNA repair proteins RtcAB regulate transcription activator RtcR via its CRISPR-associated Rossmann fold domain
Source: iScience. 2022 Oct 20;25(11):105425. doi: 10.1016/j.isci.2022.105425 (PMC9650030; doi:10.1016/j.isci.2022.105425)
Supplement: Document S1. Figures S1–S14, Tables S1–S14, and Data S1–S3 [file mmc1.pdf]

iScience, Volume *25*

Supplemental information

The RNA repair proteins RtcAB regulate transcription activator RtcR via its CRISPR-associated Rossmann fold domain

Ioly Kotta-Loizou, Maria Grazia Giuliano, Milija Jovanovic, Jorrit Schaefer, Fuzhou Ye, Nan Zhang, Danai Athina Irakleidi, Xiaojiao Liu, Xiaodong Zhang, Martin Buck, and Christoph Engl

Table S1. Genetic lesions affecting P$_{rtcBA}$-*lacZ* promoter activity and *rtc* mRNA expression levels, Related to Table 1.

		P$_{rtcBA}$-*lacZ*									
		rtc wild-type	Δ*rtcR*	Δ*rtcR* + pBAD18(*rtcR*)	Δ*rtcB*	Δ*rtcB* + pBAD18(*rtcB*)	Δ*rtcB* + pBAD18(*rtcB*$_{H337A}$)	Δ*rtcA*	Δ*rtcA* + pBAD18(*rtcA*)	chromosomal *lacZ*	*rtc* qPCR
genetic lesion	*gor*	↑↑	=	↑↑	=	↑↑	=	↑↑		=	↑
	yobF	↑↑	=	↑↑	=	↑↑	=	↑↑		=	↑
	mazF	↑↑	=	=	=	=		=	=	=	↑
	ybaK	↑↑	=	=	=	=		=	=	↓	↑
	rppH	↑	↑		↑			↑			↑
	rnhA	↑↑	↑↑		↑↑			↑↑			↑
	hfq (LB)	↑↑	↑↑		↑↑			↑↑			↑
	hfq (M9)	↓↓	↓↓		↓↓			↓↓			↓
	ahpC	↑↑	=		=			=			↑
	srmB	↑								=	↑

↑ induction (P-value<0.05)
↑↑ induction (P-value<0.001)
↓ repression (P-value<0.05)
↓↓ repression (P-value<0.001)
= no change (compared to wild-type)

Table S2. Genes affecting P$_{rtcBA}$-*lacZ* promoter activity and *rtc* mRNA expression, Related to Table 1.

		P$_{rtcBA}$-lacZ				constitutive GFP	*rtc* qPCR	genetic lesion
		LB		M9				
		rtc wild-type	Δ*rtcR*	*rtc* wild-type	Δ*rtcR*			
overexpressed gene	*yheS*	↓	=	↓	=	=	↓	=
	rof	↑	↑	↑	=	=	↑	=
	yedV	↑	=	↑	=	↓	↑	=
	fpr	↓	=	↓	=	=	=	=
	ftsX	↓	=	=	=	=	=	
	pbl'	↓	=	↓	=	=	↑	=
	yafV	↑	↑	↑	↑	=		=
	ymfI	↑	↑	↑	↑	=		=
	iadA	↓	=	=	=	↓		=
	yfaS'	↓	=	↓	=	↓		=

↑ induction (P-value<0.05)
↓ repression (P-value<0.05)
= no change (compared to wild-type)

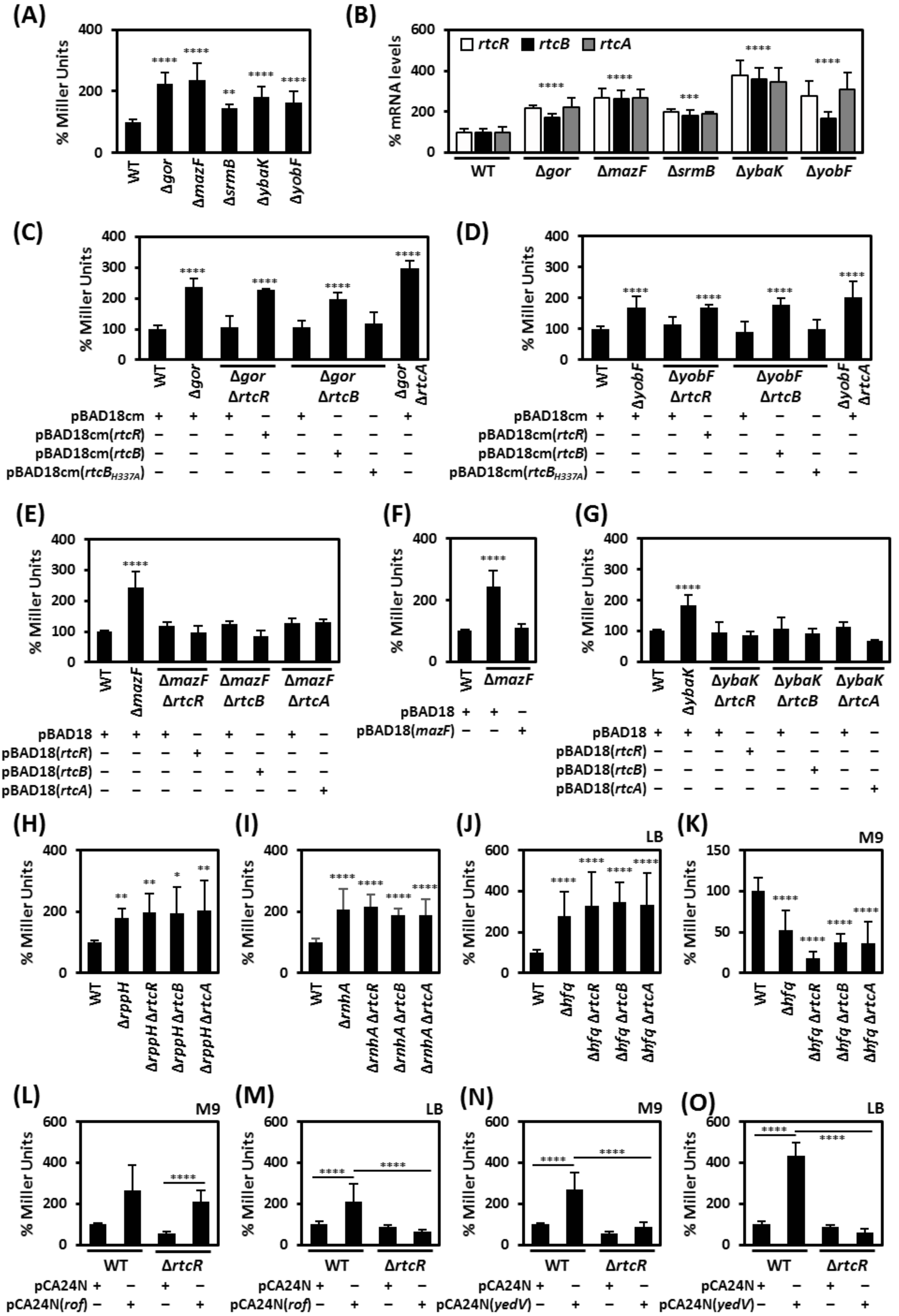

Figure S1. Regulation of the Rtc system, Related to Table 1. (A) The *rtcBA* promoter activity is induced (*N*=15) and **(B)** the *rtc* mRNA levels are increased (*N*=3) in the gene deletion mutants Δ*gor*, Δ*mazF*, Δ*srmB*, Δ*ybaK* and Δ*yobF*, compared to the wild-type strain. Both RtcR and RtcB expression from the pBAD18cm vector are required for *rtcBA* promoter activity in the gene deletion mutants **(C)** Δ*gor* (*N*=6) and **(D)** Δ*yobF* (*N*=8). RtcR, RtcB and RtcA expression

from the pBAD18cm vector is required for *rtcBA* promoter activity in the gene deletion mutants **(E)** Δ*mazF* (*N*=9) and **(G)** Δ*ybaK* (*N*=9). The effects of the *rtc* gene deletion mutants on the *rtcBA* promoter activity in cells already lacking **(E)** Δ*mazF* and **(G)** Δ*ybaK* cannot be complemented by overexpressed Rtc proteins. **(F)** The effect of the gene deletion mutant Δ*mazF* on the *rtcBA* promoter activity can be complemented with overexpressed MazF protein from the pBAD18cm vector (*N*=9). The *rtcBA* promoter activity is increased in an RtcR-independent manner by the gene deletion mutants **(H)** Δ*rppH* (*N*=4), **(I)** Δ*rnhA* (*N*=9) and **(J)** Δ*hfq* in complete medium (*N*=6), while the *rtcBA* promoter activity is repressed by **(K)** Δ*hfq* in minimal medium (*N*=4). The latter is the only identified gene deletion mutant acting as both an inducer of the Rtc system in rich media, and as a repressor in minimal media. Overexpression of the *rof* (*N*=9) and *yedV* (*N*=10) genes from the pCA24N vector results in an induction of the *rtcBA* promoter activity in **(L,N)** minimal and **(M,O)** complete medium. In all panels, beta-galactosidase activity or mRNA levels of the wild-type strain is set as 100%. Data are shown as mean and error bars represent standard deviation from the mean. *N* represents total number of independent biological replicates, with 3 technical replicates each. ANOVA * P-value < 0.05; ** P-value < 0.01; *** P-value < 0.001; **** P-value < 0.0001.

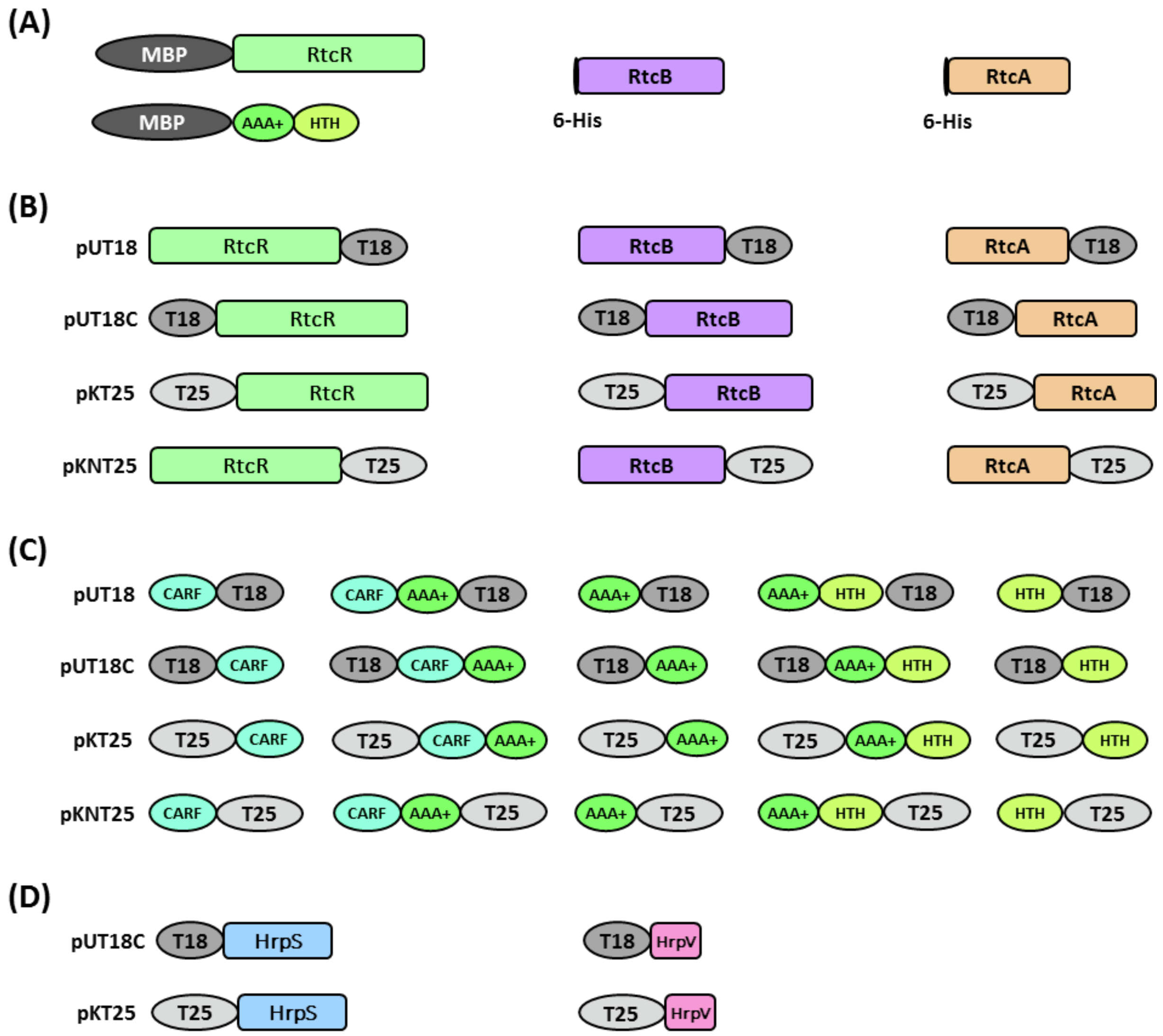

Figure S2. Schematic representation of constructs, Related to Figure 2. (A) MBP- or His-tagged purified Rtc proteins used for gel filtration chromatography and crosslinking; **(B)** T18/T25 N-terminal and C-terminal fusions of full-length Rtc proteins produced by the bacterial two-hybrid vectors; **(C)** T18/T25 N-terminal and C-terminal fusions of truncated RtcR domains produced by the bacterial two-hybrid vectors; **(D)** T18/T25 fusions of Hrp proteins produced by the bacterial two-hybrid vectors.

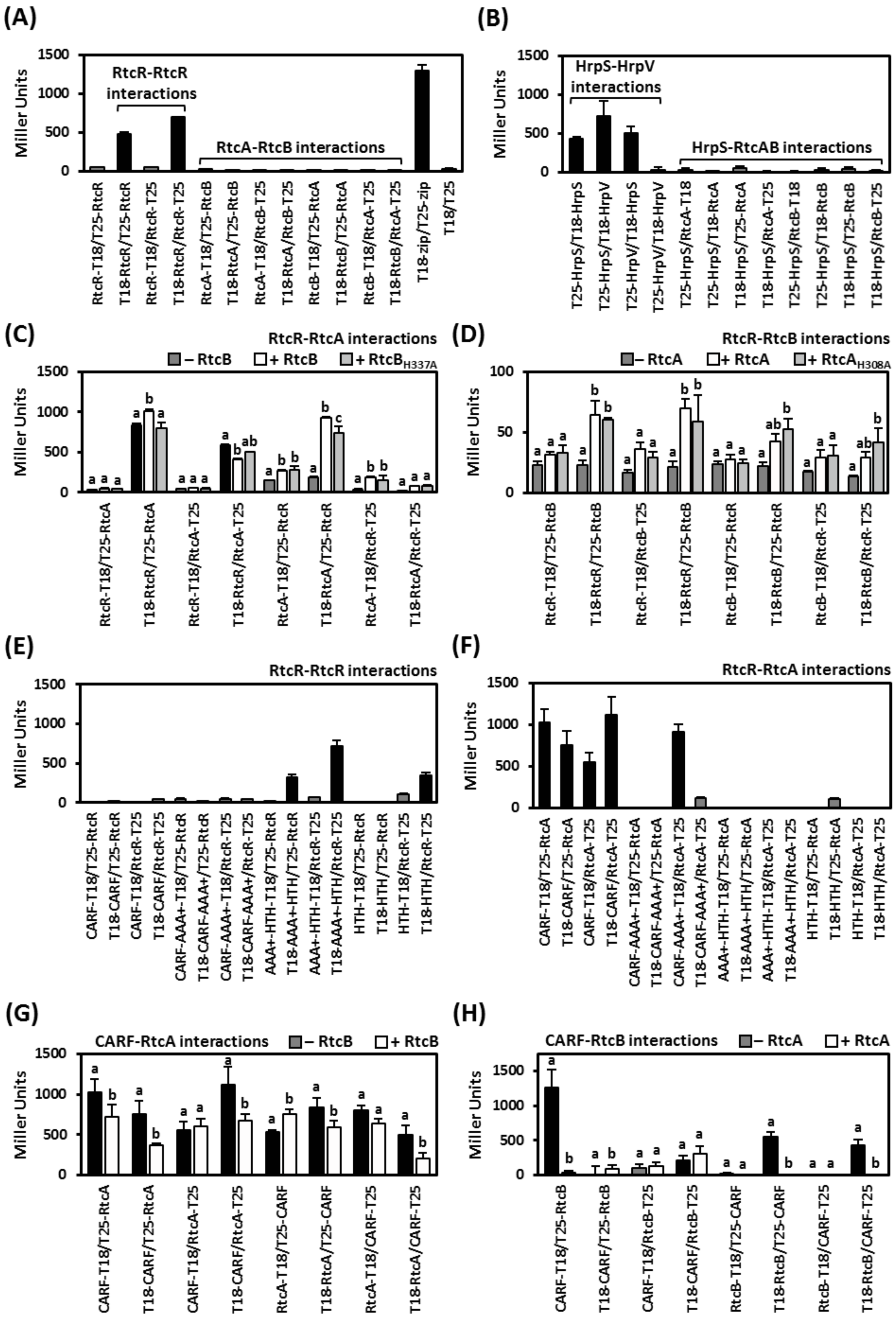

Figure S3. Interactions between Rtc proteins revealed by bacterial 2-hybrid, Related to Figure 2. (A) RtcR interacts with itself. **(B)** HrpS interacts with itself and HrpV, but not with RtcA or RtcB. **(C)** RtcR interacts with RtcA and RtcB affects their interaction. **(D)** RtcA affects the interaction between RtcR and RtcB. **(E)** The interaction between RtcR proteins is mediated via the HTH DNA binding domain at the C-terminus. **(F)** The interaction between RtcR and RtcA proteins is mediated via the CARF signalling domain at the N-terminus. **(G)** TheRtcR CARF domain interacts with RtcA and RtcB affects their interaction. **(H)** The RtcR CARF domain interacts with RtcB and RtcA affects their interaction.

Data are shown as mean and error bars represent standard deviation from the mean. *N*=3 and represents total number of independent biological replicates, with 3 technical replicates each. Black columns but not dark grey columns are significantly different (ANOVA P-value < 0.0001) as compared to the negative control (T18/T25). Different letters indicate statistically significant differences (ANOVA P-value < 0.01 at least).

Data S1. *In vitro* and *in vivo* protein crosslinking, Related to Figure 2.

In vitro glutaraldehyde protein crosslinking experiments support the pairwise interactions of RtcA and RtcB with RtcR: as estimated from image analysis of stained gels and immunoblots, the amount of high molecular weight complexes in the presence of MBP-RtcR together with His-RtcA or His-RtcB is 2-3 fold higher as compared to samples of His-RtcA, His-RtcB or MBP-RtcR alone treated with the crosslinker (Fig. S4A). This increase was not evident in the presence of MBP-RtcR$_{\Delta CARF}$ together with His-RtcA or His-RtcB (Fig. S4B). Since the interaction between RtcR and RtcB was not detected *in vivo* using the bacterial two-hybrid assay, although it was observed in all the *in vitro* experiments, a different approach was adopted: *in vivo* DSP crosslinking was performed on cells expressing MBP-RtcR and RtcB-His, separately and together, and the outcome was assessed by SDS-PAGE and immunoblotting (Fig. S4D). The levels of both proteins were lower in the samples in which they were co-expressed as compared to when they were expressed separately (Fig. S4D). When DSP was added, the quantity of both MBP-RtcR and RtcB-His detected was reduced in the presence as compared to the absence of the other Rtc protein. This reduction was reversed by the addition of DTT (Fig. S4D), suggesting that a complex may be formed between MBP-RtcR and RtcB-His. Since a single discrete new crosslinked complex was not evident by immunoblotting, it may be present as multiple dispersed crosslinked species distributed across the gel lane. Alternatively, or in addition, a high molecular weight complex unable to enter the gel or transfer may be formed. Such complexes may include cell components such as those of the ribosome that is known to interact with RtcB and RtcA (Fig. S4E; Temmel *et al.*, 2017).

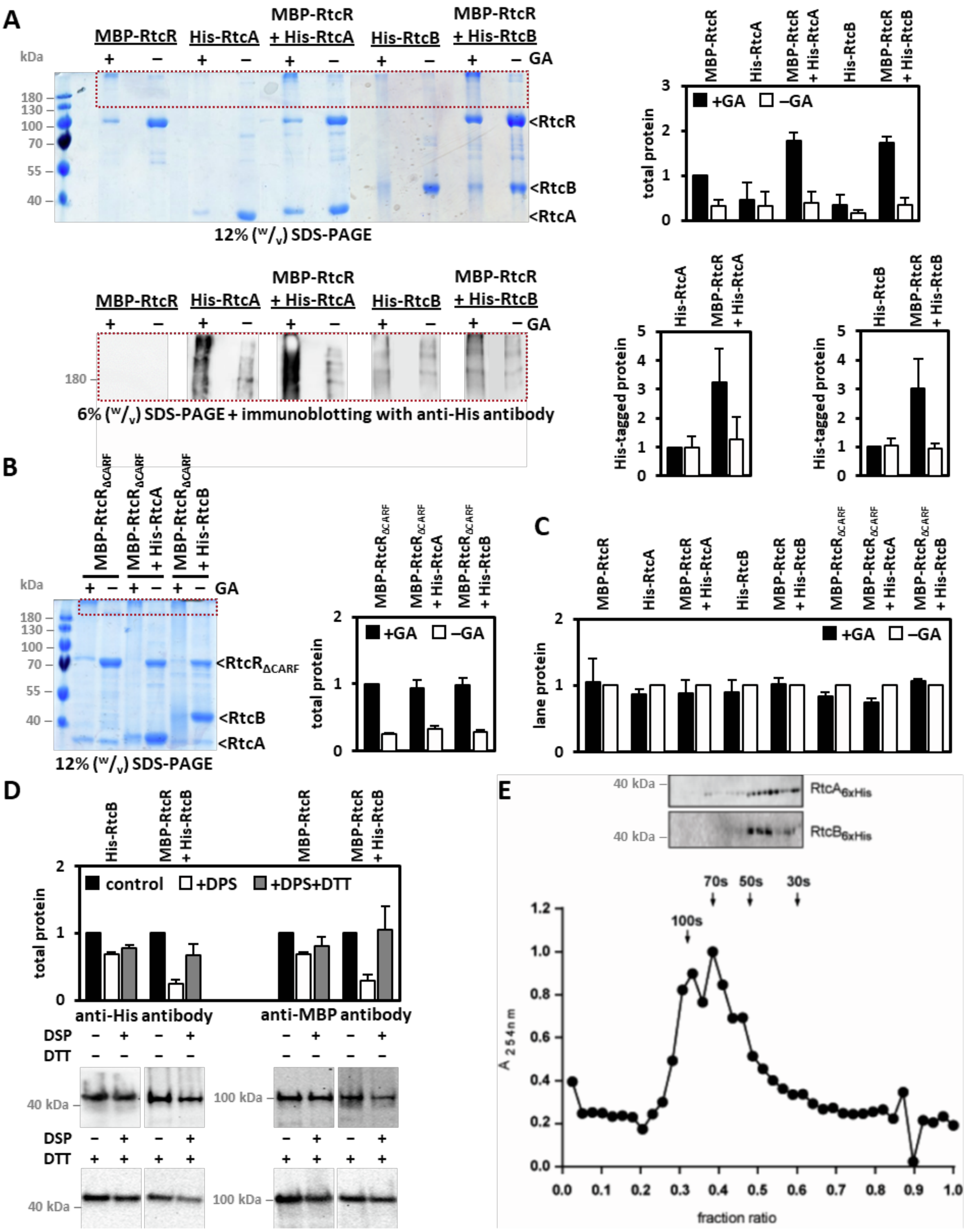

Figure S4. Interactions between Rtc proteins revealed by crosslinking Related to Figure 2. (A) Representative SDS-PAGE (top left) and immunoblotting using an anti-His antibody (bottom left) of MBP-RtcR with His-RtcA or His-RtcB, following *in vitro* protein crosslinking with glutaraldehyde (GA). Quantification using ImageJ of the total protein present in the red square following SDS-PAGE (top right); the His-RtcA and His-RtcB (bottom right) present in the red box following immunoblotting. **(B)** Representative SDS-PAGE of MBP-RtcR$_{\Delta CARF}$ with His-RtcA or His-RtcB, following *in vitro* protein crosslinking with glutaraldehyde (GA) (left). Quantification using ImageJ of the total protein present in the red box following SDS-PAGE (right). **(C)** Quantification using ImageJ of the total protein present in each lane following SDS-PAGE. **(D)** Representative immunoblotting using an anti-His antibody (bottom left) or anti-MBP antibody (bottom right) of respectively His-RtcB and MBP-RtcR, alone or in combination, following *in vivo* DSP crosslinking and

DTT-mediated crosslinking reversal. Quantification using ImageJ of His-RtcB and MBP-RtR following SDS-PAGE and immunoblotting. **(E)** Sucrose gradient centrifugation of *E. coli* extracts under associative ribosome profiling conditions shows that His-RtcA and His-RtcB each associate with high molecular weight complexes characteristic of ribosome assemblies. In all panels, data are shown as mean and error bars represent standard deviation from the mean of triplicates.

Table S3. List of potential RtcR RNA ligands tested for transcriptional activation, Related to Figure 3.

Type	Description
components of the translation mechanism	*E. coli* 16S rRNA, tRNA
oligonucleotides	cyclic tetra/hexa adenylates, individually and together<br>linear tetra adenylates<br>linear tetra nucleotides (random)
crude cell extracts of *E. coli* cells	WT, Hpx-, Δ*gor*, Δ*srmB*; 8 and 24 hpi

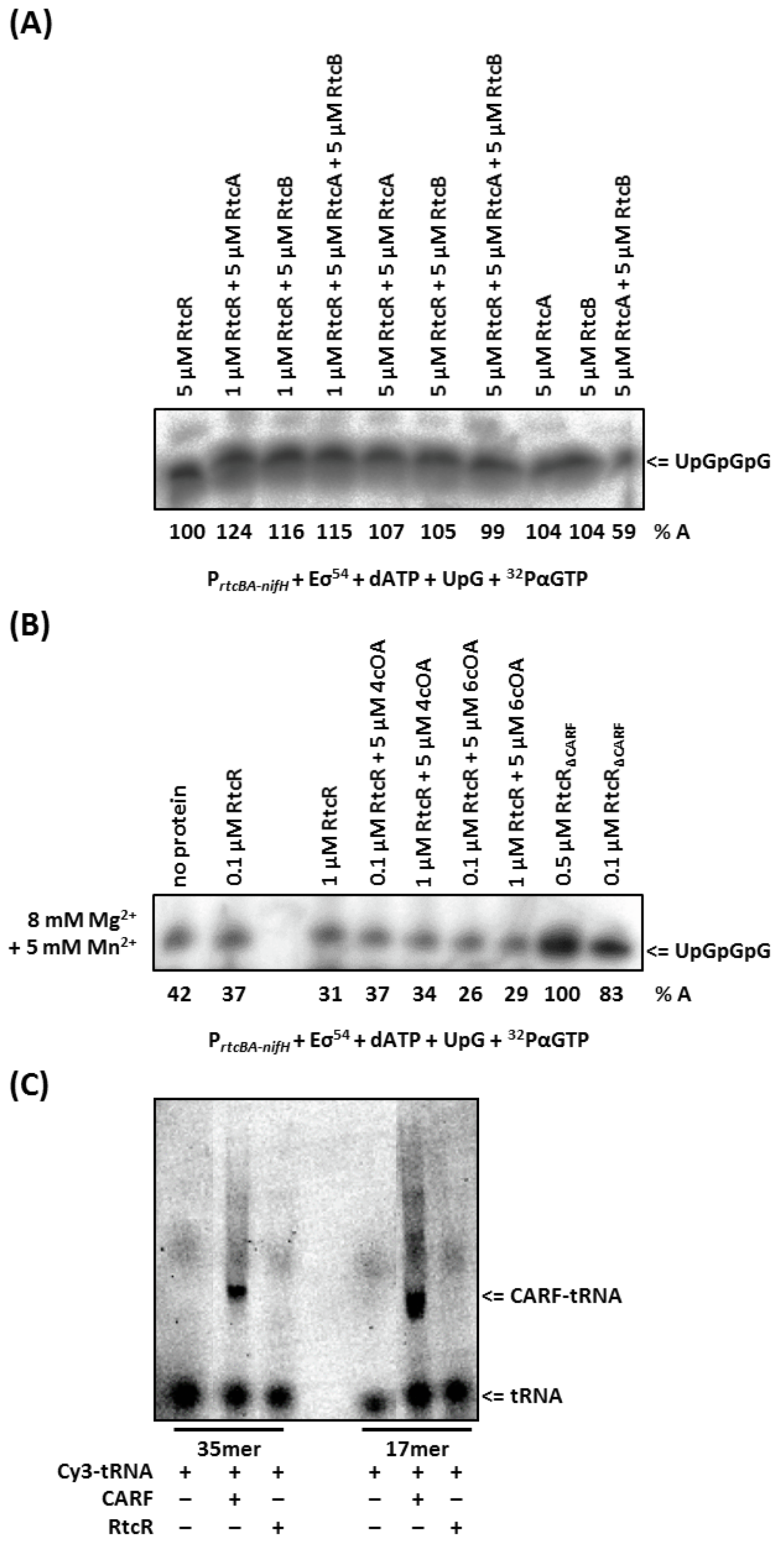

Figure S5. Potential RtcR ligands and activators, Related to Figure 3. UpGpGpG spRNAs were electrophoresed on 20% ($^w/_v$) urea-PAGE following production from the super-coiled $P_{rtcBA-nifH}$ hybrid promoter template in the presence of **(A)** RtcA and/or RtcB; **(B)** Mg^{2+} and Mn^{2+}, on their own or with cyclic tetra/hexa (4/6) adenylates (cOA). % A represents percentage of activity. **(C)** Fragments of Cy3-$tRNA^{Glu(UUC)}$ were electrophoresed on 4% ($^w/_v$) PAGE in the presence of the RtcR CARF domain and full length RtcR.

Table S4. SNPs in Hpx- compared to wild-type MG1655, Related to Figure 4.

SNP	Position in chromosome	Gene	Type of mutation
AC	223606	N/A	N/A
CG	271633	*insI1* (protein coding)	non-synomynous (Ala = > Arg)
GC	271632		
TC	275022	*insH1* (protein coding)	synonymous
CT	736682	*ybfQ* (protein coding)	stop codon
CT	736663		synonymous
CT	1097158	N/A	N/A
GA	1180931	*ycfZ* (protein coding)	stop codon
CT	1299511	*oppA5'* (sRNA coding)	N/A
AG	1300681		
GA	1836397	*ynjB* (protein coding)	non-synomynous (Glu = > Lys)
CT	1848300	*ynjA* (protein coding)	non-synomynous (Gly = > Lys)
CT	1848301		
GA	2620956	N/A	N/A
AC	3446246	*rpsH* (protein coding)	synonymous
CT	4093770	*rhaD* (protein coding)	non-synomynous (Gly = > Asp)
GT	4326288	N/A	N/A

Table S5. Gene deletion mutants associated with oxidative stress, Related to Figure 4.

Name	Description	Rtc induction
ΔahpC[1]	Peroxidase; forms alkyl hydroperoxide reductase with peroxiredoxin reductase *ahpF*	YES
Δfur	Iron responsive regulator; transcription activation of *katE*, *katG* etc	NO
Δgor	GSH oxidoreductase	YES
ΔiscR	Iron-sulfur cluster regulator	NO
ΔkatE	Monofunctional catalase HPII	NO
ΔkatG	Bifunctional catalase HPI	NO
ΔoxyR	Oxidative stress regulator; transcription activation of *ahpCF*, *fur*, *gor*, *katG* etc	NO

[1] responsible for detoxifying the majority of H_2O_2 in the cells (Winterbourn, 2008)

Table S6. Predicted effects of cysteine mutations on the RtcR CARF domain, Related to Figure 4.

	**Missense 3D**		**Predicted stability change (ΔΔG)**			
	**Structural damage**	**RMSD***	**mCSM**	**SDM**	**DUET**	**RMSD***
**C32A**	buried / exposed switch	0.00	−1.388	−0.470	−1.151	0.01
**C34A**	none	0.00	−1.820	−0.550	−1.795	0.00
**C91A**	none	0.00	−1.482	+0.795	−0.898	0.01
**C122A**	buried H-bond breakage	0.00	−1.102	−0.440	−0.831	0.01

* as compared to the *in silico* structural model of the RtcR CARF domain (Fig. S6)

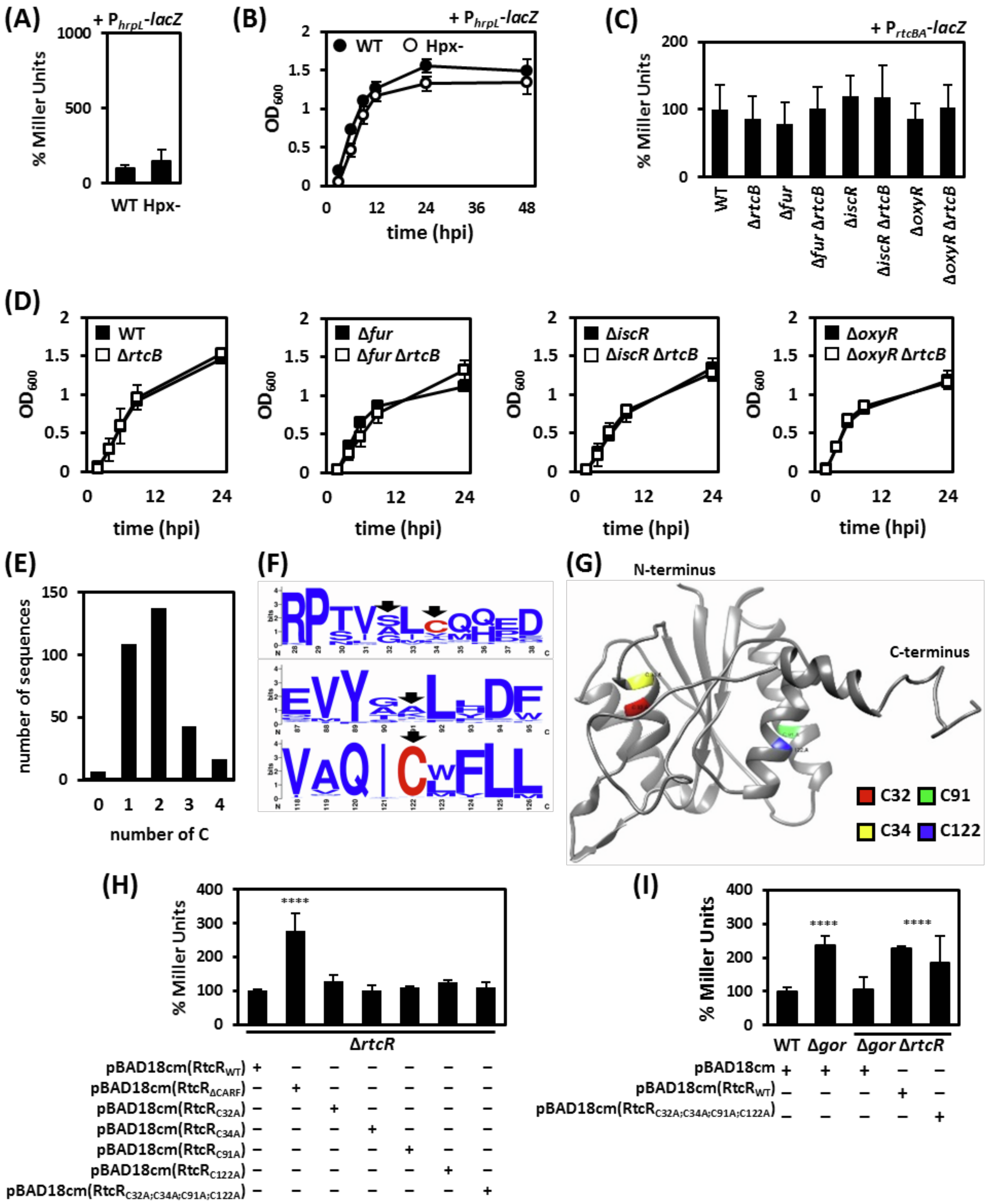

Figure S6. The Rtc system and oxidative stress, Related to Figure 4. (A) The negative control test *hrp* promoter activity is not induced in the Hpx- strain after 24 h (*N*=3) and **(B)** growth of the Hpx- strain compared to the wild-type is not inhibited by the presence of the P_{hrp}-*lacZ* reporter plasmid (*N*=3). **(C)** The *rtcBA* promoter activity is not induced by the gene deletion mutants Δ*fur*, Δ*iscR* and Δ*oxyR* in minimal medium after 24 h (*N*=3) and **(D)** growth of the Δ*fur*, Δ*iscR* and Δ*oxyR* strains is not inhibited by deletion of the *rtcB* gene (*N*=3). **(E)** The 314 CARF domain sequences in Pfam contain a number of cysteine residues ranging from zero to four. **(F)** Two of the cysteine residues are conserved among the aligned 314 CARF domain sequences as illustrated by the weblogo; arrows indicate the positions of the four cysteines in the *E. coli* RtcR CARF domain. **(G)** The *E. coli* RtcR CARF domain cysteine residues could potentially form disulphide bridges based on the *in silico* structural model. **(H)** Site-directed mutagenesis of the RtcR CARF domain cysteine residues into alanine residues does not affect the repression of the *rtcBA* promoter activity under non inducing conditions (*N*=4). **(I)** Site-directed mutagenesis of the RtcR CARF domain cysteine residues into alanine

residues does not affect the activation of the *rtcBA* promoter activity under inducing conditions (*N*=3). In all panels, beta-galactosidase activity of the wild-type strain is set as 100%. Data are shown as mean and error bars represent standard deviation from the mean. *N* represents total number of independent biological replicates, with 3 technical replicates each. ANOVA **** P-value < 0.0001.

Data S2. Transcriptome profiling of Rtc inducing conditions, Related to Figure 5.

In all cases, quality assessment revealed that the majority of reads (> 80%) correspond to known features of the annotated *E. coli* genome (Fig. S7A), while the gene deletion mutants and the induction of the Rtc system as judged by *rtcBA* up regulation were also verified (Fig. S7B).

In *E. coli* cells lacking *gor* or *mazF*, 30 and 33 genes, respectively were differentially expressed as compared to the wild-type. In total, 24 of these transcripts were common to both gene deletion mutants (Fig. 5A) and the vast majority were protein-coding. Decreases in the expression levels of genes forming the glycerol-3-phosphate dehydrogenase complex were observed (Fig. S8B), potentially due to increases in the expression levels of *glpR*, the glycerol-3-phosphate regulon repressor located adjacent to the Rtc system on the *E. coli* chromosome. This link was also evident following GO enrichment analysis (Tables S7,S8). Furthermore, the Psp membrane stress response protein transcripts were increased in abundance and the system responsible for arabinose catabolism was affected (Fig. S8D).

Only 3 transcripts were significantly more abundant in *E. coli* cells lacking *srmB*: the outer membrane autotransporter *flu* and the small RNA *isrC*, whose expression is inhibited by OxyR, together with the tRNA for aspartic acid. Valine tRNA was also more abundant but this was not statistically significant potentially due to discrepancies between the replicates.

A very large number, up to 40% of the total *E. coli* genes, were differentially expressed in Hpx-[RtcON] and Hpx-[RtcOFF] as compared to the wild-type: 699 and 1777 respectively 8 h post-inoculation, and 1144 and 460 respectively 24 h post-inoculation. In all cases the vast majority (over 85%) of the differentially expressed transcripts encode proteins (Fig. S7C). Principal component analysis revealed that the major source of variation (PC1) among the samples is time of harvest, while the second major source or variation (PC2) is the genetic background of the strains used (Fig. 5B). All wild-type strains group together, while Hpx-[RtcON] and Hpx-[RtcOFF] were distinct both from the wild-type and from each other (Fig. 5B), potentially due to the lack of *rtcBA* expression in the latter.

GO enrichment analysis of the differentially expressed transcripts revealed overrepresentation of genes forming the glycerol-3-phosphate dehydrogenase complex in Hpx-[RtcON] both at 8 (Table S9) and 24 h post-inoculation (Cellular Component 'glycerol-3-phosphate dehydrogenase complex'; fold enrichment 6.28; p-value 2.34·10-02), in agreement with that was observed in the cells lacking *gor* and *mazF* (Tables S7,S8), and Hpx-[RtcOFF] (Fig. S8B). KEGG pathway analysis also showed an effect on carbohydrate and lipid metabolism (Tables S11,12). Similarly, and possibly linked to changes in lipid metabolism, the Psp membrane stress response protein transcripts were more abundant in both Hpx-[RtcON] and Hpx-[RtcOFF] (Fig. S8D).

Additionally, both GO enrichment and KEGG pathway analysis illustrated an effect on ribosomal proteins, most prominent in the genes down regulated in Hpx-[RtcON] at 8 h post-inoculation (Tables S9,S11). Indeed, 25/31 and 14/21 proteins of the large and small ribosomal subunits, respectively, were significantly regulated in Hpx- at 8 h post-inoculation (Fig. S9). A similar trend was noted in Hpx-[RtcON] (Cellular Component 'cytosolic large ribosomal subunit'; fold enrichment 4.02; p-value 2.23·10-02) and Hpx-[RtcOFF] (Cellular Component 'ribosomal subunit'; fold enrichment 2.33; p-value 4.28·10-02 and 'cytosolic ribosome'; fold enrichment 2.33; p-value 3.98·10-02) at 24 h post-inoculation (Table S11). In contrast, GO enrichment and KEGG pathway analysis did not reveal any significant overall effect on ribosomal proteins in Hpx-[RtcOFF] at 8 h post-inoculation, even though individual genes are up regulated or down regulated (Fig. S9). The down regulation of ribosomal proteins and the constituents of the bacterial-type flagellum

hook at 8 h post-inoculation was notable exclusively in Hpx-[RtcON] but not Hpx-[RtcOFF] (Table S10). However, an effect on nucleotide metabolism (Table S11) and a global down regulation of ribosomal RNAs (Fig. 5D) was observed in Hpx-[RtcOFF] at 8 h post-inoculation but not in any other condition.

Finally, the expression levels of genes whose deletion or overexpression modulates the RtcR system was not significantly affected in *E. coli* cells lacking *rtcR*, but some were differentially regulated in *E. coli* cells lacking *rtcA* or *rtcB* (Fig. S11) and their numbers were significantly higher than those expected by chance ($P < 0.0001$ and $P < 0.01$ for cells lacking *rtcA* or *rtcB*, respectively). Although evidence for feedback loops affecting RtcAB expression arose from examining the relationships between *rtc* expression and particular gene deletions and over expressions, but these relationships are complex and not fully explained and there was no discernible pattern associating the expression levels of the genes with their regulatory effect on *rtc* or their function; e.g. there are (1) genes, such as those related to oxidative stress including the alkyl hydroperoxide reductase component *ahpC*, whose deletion induces Rtc and *rtc* deletion increases their expression; (2) genes, including the ribotoxin *mazF* and the ribonuclease *rnhA*, whose deletion induces Rtc and *rtc* deletion decreases their expression; (3) genes, such as the transcription antiterminator *rof*, whose overexpression induces Rtc and *rtc* deletion increases their expression; (4) genes, such as the histidine kinase *yedV*, whose overexpression induces Rtc and *rtc* deletion decreases their expression; and (5) genes, such as the energy-dependent translational throttle protein *ettA* (formerly *yjjK*), whose overexpression represses Rtc and *rtc* deletion increases their expression.

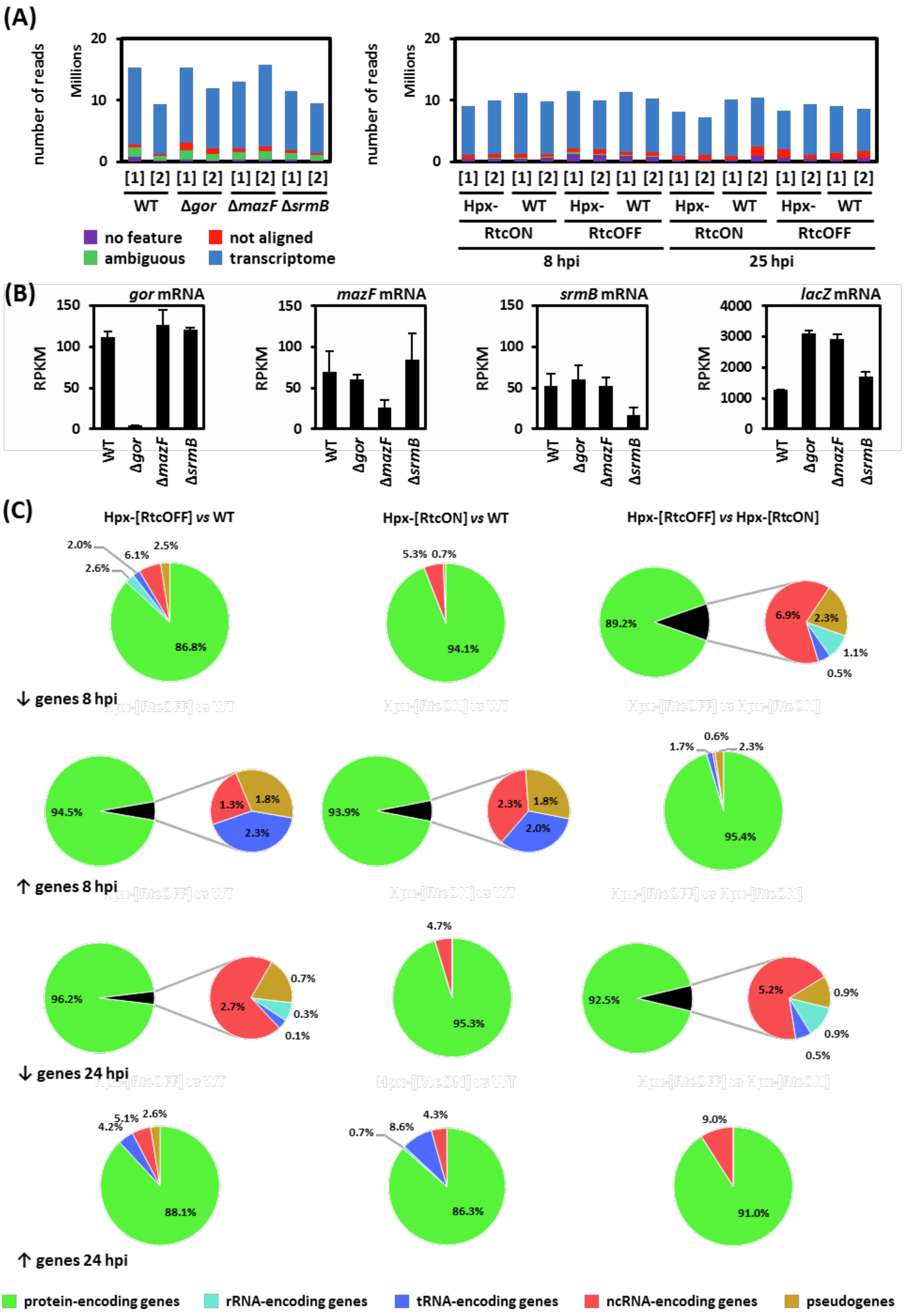

Figure S7. NGS quality assessment and initial analysis, Related to Figure 5. (A) The majority of reads (> 80%) mapped to the annotated *E. coli* MG1655 genome, as calculated by HTSeq. **(B)** The gene deletion mutants *gor*, *mazF* and *srmB* have lower RPKM values compared to the wild-type. The *lacZ* mRNA, produced by the P_{rtcBA}-*lacZ* reporter plasmid and therefore corresponding to *rtcBA* induction, has a higher RPKM value in the gene deletion mutants compared to the

wild-type. Data are shown as mean and error bars represent standard deviation from the mean. **(C)** Pie charts illustrating the general functional roles of genes differentially expressed in Hpx-[RtcON] and Hpx-[RtcOFF] compared to the wild-type *E. coli* and to each other at 8 and 24 hours post inoculation.

Table S7. GO enrichment analysis of genes differentially expressed in *E. coli* cells lacking *gor*, Related to Figure 5.

	percentage of genes	fold enrichment	P-value
**Biological Process**			
glycerol-3-phosphate catabolic process	13.3%	> 100	$8.96 \cdot 10^{-05}$
arabinose catabolic process	10.0%	63.74	$2.53 \cdot 10^{-02}$
**Molecular Function**			
glycerol-3-phosphate dehydrogenase (quinone) activity	13.3%	> 100	$7.75 \cdot 10^{-05}$
**Cellular Component**			
glycerol-3-phosphate dehydrogenase complex	13.3%	> 100	$1.28 \cdot 10^{-05}$

Table S8. GO enrichment analysis of genes differentially expressed in *E. coli* cells lacking *mazF*, Related to Figure 5.

	percentage of genes	fold enrichment	P-value
**Biological Process**			
glycerol-3-phosphate catabolic process	12.1%	> 100	$1.53 \cdot 10^{-04}$
**Molecular Function**			
glycerol-3-phosphate dehydrogenase (quinone) activity	12.1%	> 100	$1.33 \cdot 10^{-04}$
**Cellular Component**			
glycerol-3-phosphate dehydrogenase complex	12.1%	> 100	$2.19 \cdot 10^{-05}$

Table S9. GO enrichment analysis of genes expressed in Hpx-[RtcON] cells 8 hpi, Related to Figure 5.

	percentage of genes	fold enrichment	P-value
**Biological Process**			
ribosomal large subunit assembly	9.5%	6.73	$4.99 \cdot 10^{-04}$
**Molecular Function**			
structural constituent of ribosome	8.5%	7.57	$4.73 \cdot 10^{-09}$
rRNA binding	3.9%	6.40	$4.73 \cdot 10^{-03}$
**Cellular Component**			
glycerol-3-phosphate dehydrogenase complex	1.4%	15.13	$8.04 \cdot 10^{-03}$
bacterial-type flagellum hook	1.4%	8.65	$3.14 \cdot 10^{-02}$
cytosolic large ribosomal subunit	6.4%	8.51	$6.70 \cdot 10^{-09}$
cytosolic small ribosomal subunit	2.8%	4.84	$7.36 \cdot 10^{-03}$

Table S10. GO enrichment analysis of genes differentially expressed in Hpx-[RtcON] but not Hpx-[RtcOFF] cells 8 hpi, Related to Figure 5.

	percentage of genes	fold enrichment	P-value
**Biological Process**			
ribosomal large subunit assembly	4.0%	6.34	$6.00 \cdot 10^{-03}$
**Molecular Function**			
structural constituent of ribosome	8.0%	7.13	$5.47 \cdot 10^{-07}$
**Cellular Component**			
NarGHI complex	1.2%	17.12	$4.62 \cdot 10^{-02}$
bacterial-type flagellum hook	1.6%	9.78	$3.69 \cdot 10^{-02}$
cytosolic large ribosomal subunit	6.0%	8.02	$3.44 \cdot 10^{-07}$

Table S11. KEGG pathway analysis of the genes differentially regulated in Hpx- cells 8 hpi, Related to Figure 5.

KEGG pathway		Hpx-[RtcON] *vs* WT				Hpx-[RtcOFF] *vs* WT			
		down-regulated	up-regulated	total	P-value	down-regulated	up-regulated	total	P-value
metabolism	carbohydrates	33	42	75	< 0.01	81	68	149	<0.001
	energy	6	23	29	NS	20	27	47	NS
	lipids	13	3	16	NS	17	13	30	NS
	nucleotides	6	7	13	NS	15	39	54	< 0.05
	amino acids	25	16	41	NS	55	43	98	NS
	glycans	2	1	3	NS	8	12	20	NS
	cofactors and vitamins	7	19	26	NS	22	55	77	NS
	terpenoids and polyketides	3	1	4	NS	1	13	14	NS
	secondary	3	2	5	NS	2	6	8	NS
	xenobiotics	6	0	6	NS	9	4	13	NS
**Transcription**		2	0	2	NS	2	1	3	NS
**Translation**		28	0	28	<0.001	3	20	23	NS
**Folding, sorting and degradation**		2	5	7	NS	4	19	13	NS
**Replication and repair**		1	3	4	NS	4	16	18	NS
**Membrane transport**		18	29	47	NS	48	39	87	NS
**Signal transduction**		15	22	37	NS	31	21	52	NS
**Cellular community**		16	6	22	NS	34	15	49	NS
**Cell motility**		15	2	17	<0.05	10	3	13	NS
**Drug resistance: antimicrobial**		9	4	13	NS	11	8	19	NS

Table S12. KEGG pathway analysis of the genes differentially regulated in Hpx- cells 24 hpi, Related to Figure 5.

KEGG pathway		Hpx-[RtcON] *vs* WT				Hpx-[RtcOFF] *vs* WT			
		down-regulated	up-regulated	total	P-value	down-regulated	up-regulated	total	P-value
metabolism	carbohydrates	31	4	35	NS	57	39	96	<0.05
	energy	11	8	19	NS	26	10	36	NS
	lipids	31	0	31	<0.0001	22	3	25	< 0.05
	nucleotides	6	4	10	NS	13	11	24	NS
	amino acids	32	7	39	NS	55	23	78	NS
	glycans	6	0	6	NS	6	0	6	NS
	cofactors and vitamins	19	4	23	NS	42	11	53	NS
	terpenoids and polyketides	4	0	4	NS	7	6	13	< 0.05
	secondary	1	2	3	NS	2	2	4	NS
	xenobiotics	3	0	3	NS	5	1	6	NS
**Transcription**		0	0	0	NS	1	0	1	NS
**Translation**		15	7	22	<0.01	31	9	40	<0.0001
**Folding, sorting and degradation**		6	0	6	NS	16	4	20	NS
**Replication and repair**		6	1	7	NS	12	2	14	NS
**Membrane transport**		19	12	31	NS	41	20	61	NS
**Signal transduction**		3	2	5	NS	8	11	19	NS
**Cellular community**		10	6	16	NS	22	3	25	NS
**Cell motility**		2	2	4	NS	2	9	11	NS
**Drug resistance: antimicrobial**		4	0	4	NS	11	3	14	NS

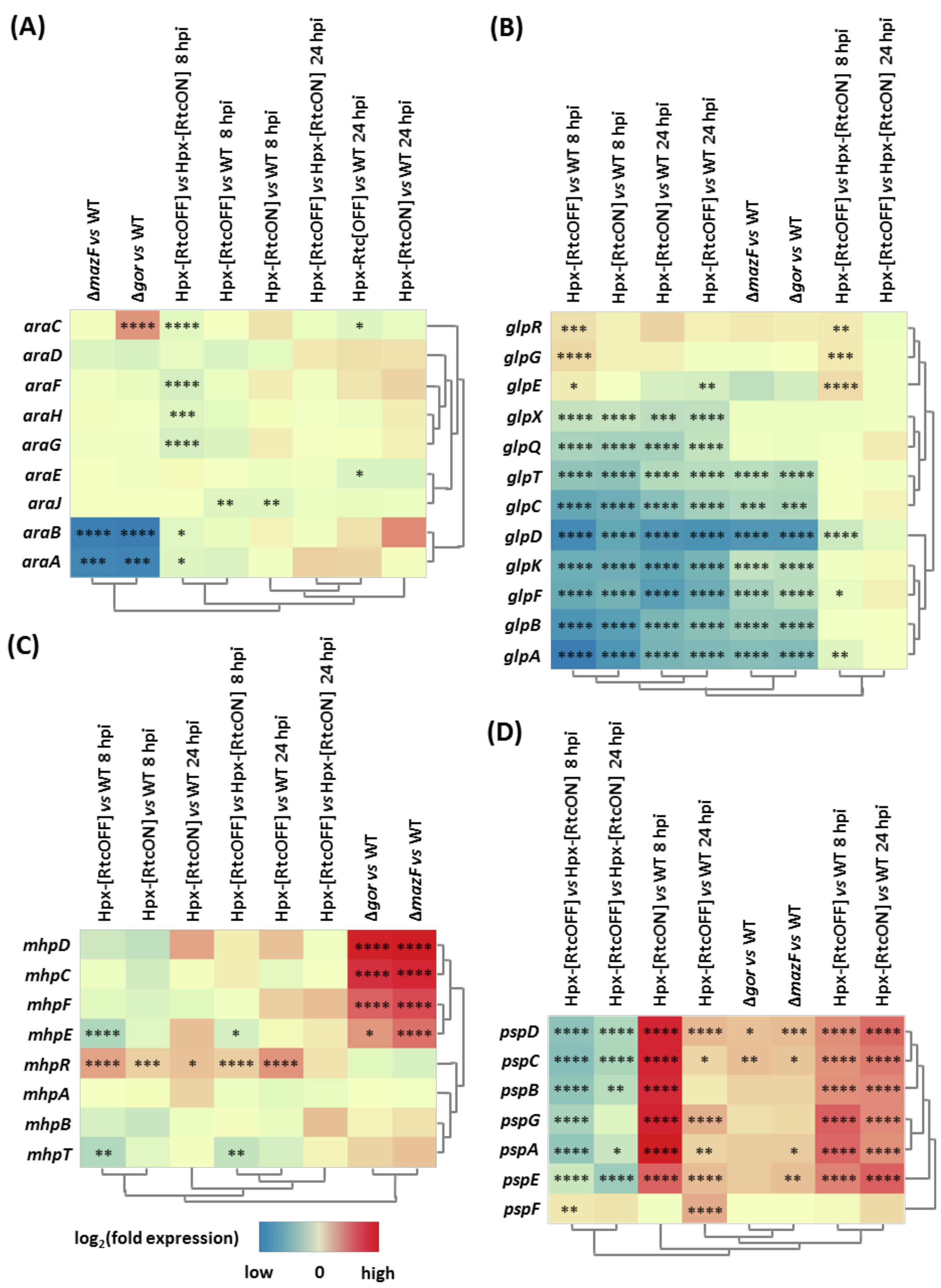

Figure S8. Expression of selected systems affected under Rtc-inducing conditions, Related to Figure 5. Heat map of expression levels of genes belonging to **(A)** the arabinose (*ara*) system (log_2fold expression ±9), **(B)** the glycerol-3-phosphate (*glp*) system (log_2fold expression ±9), **(C)** the m-hydroxyphenylpropionate (*mhp*) system (log_2fold expression ±6) and **(D)** the *psp* membrane stress response system (log_2fold expression ±8) in *E. coli* cells lacking *gor* or *mazF* and in the Hpx- strain, as shown by NGS. Rows and columns have been grouped based on a hierarchical clustering algorithm. Limma/DESeq2 * adjusted P-value < 0.05; ** adjusted P-value < 0.01; *** adjusted P-value < 0.001; **** adjusted P-value < 0.0001.

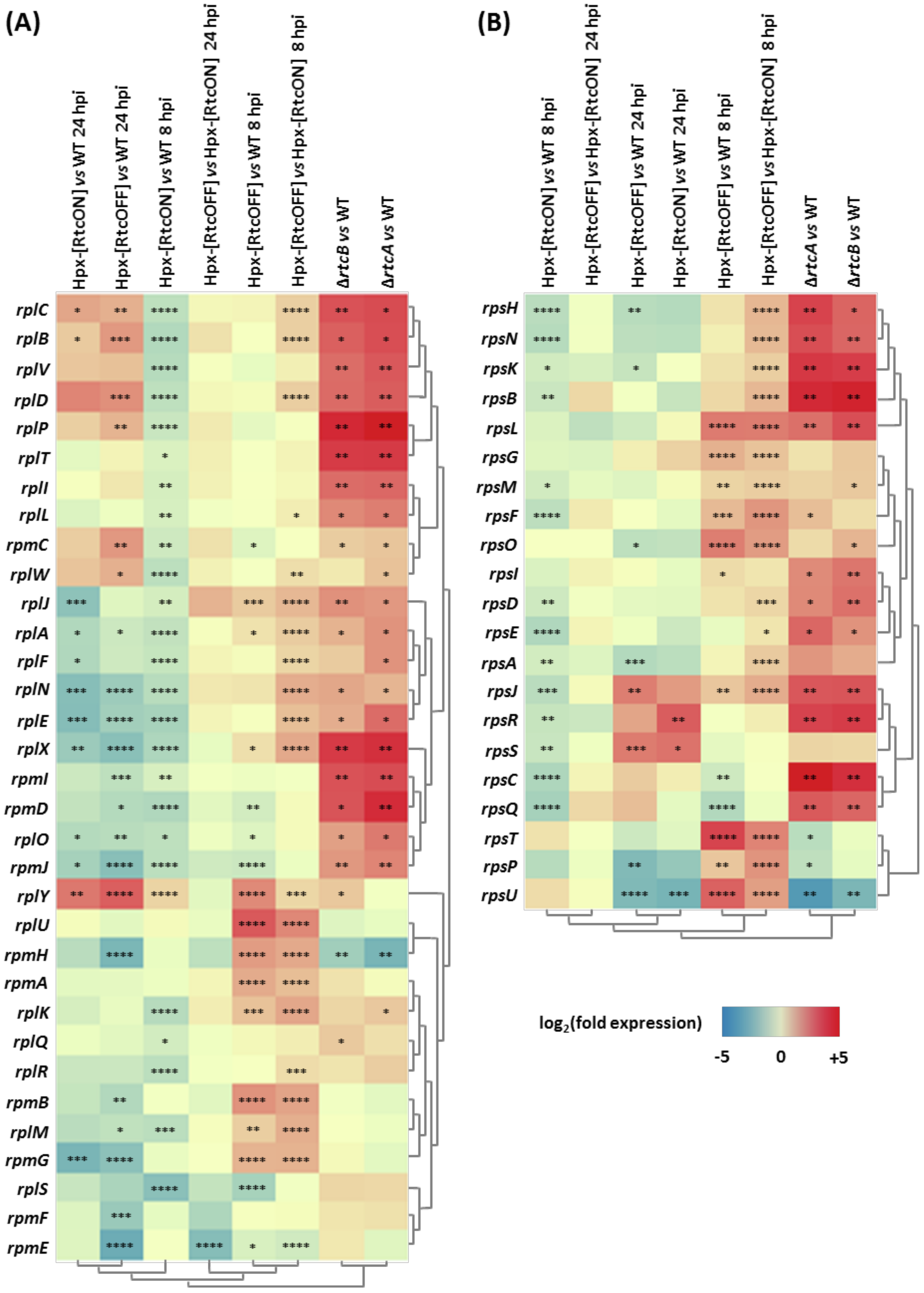

Figure S9. Expression of ribosomal proteins, Related to Figure 5. Heat map of expression levels of genes encoding ribosomal proteins for **(A)** the large and **(B)** the small ribosomal subunit in *E. coli* cells lacking *rtc* and in the Hpx- strain, as shown by NGS. Rows and columns have been grouped based on a hierarchical clustering algorithm. Limma/DESeq2 * adjusted P-value < 0.05; ** adjusted P-value < 0.01; *** adjusted P-value < 0.001; **** adjusted P-value < 0.0001.

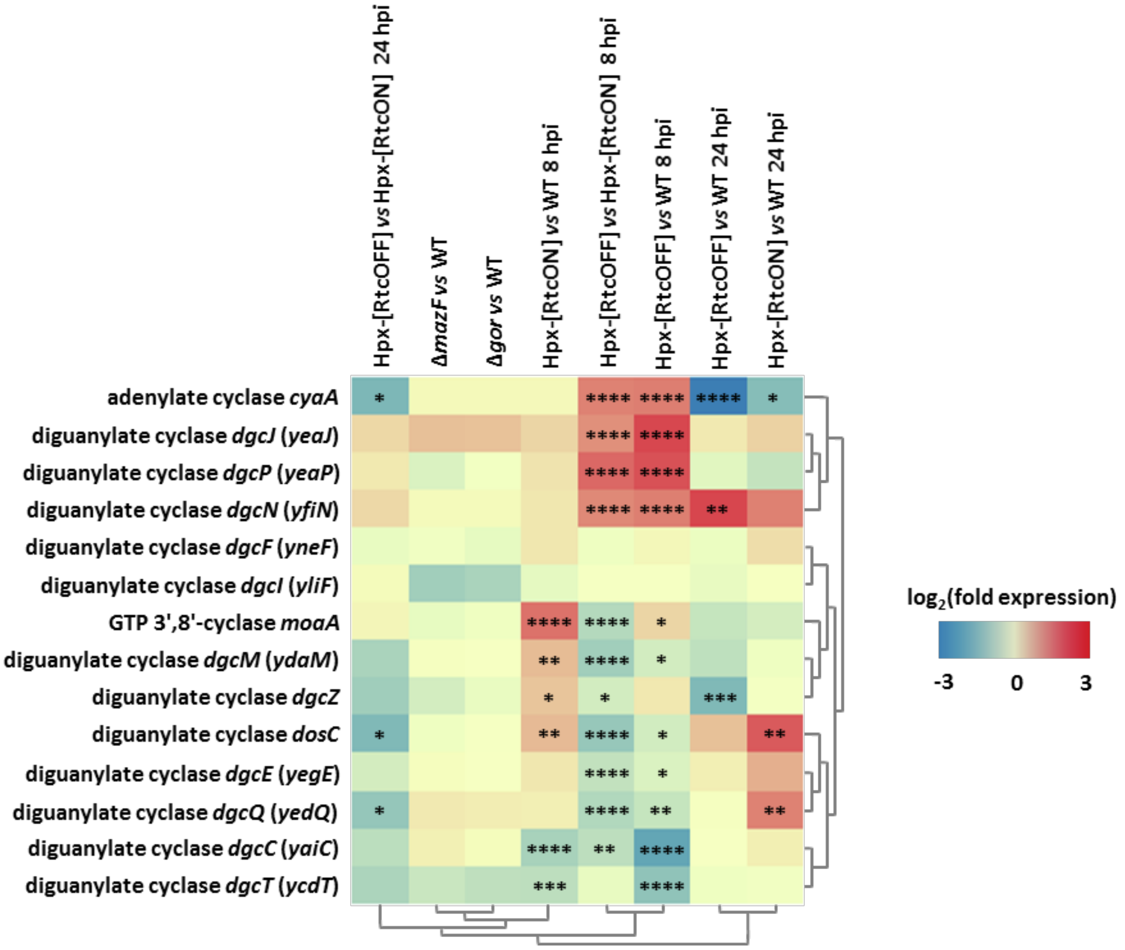

Figure S10. Expression of cyclases under Rtc-inducing conditions, Related to Figure 5. Heat map of expression levels of genes encoding cyclases in *E. coli* cells lacking *gor* or *mazF* and in the Hpx- strain, as shown by NGS. Rows and columns have been grouped based on a hierarchical clustering algorithm. DESeq2 * adjusted P-value < 0.05; ** adjusted P-value < 0.01; *** adjusted P-value < 0.001; **** adjusted P-value < 0.0001.

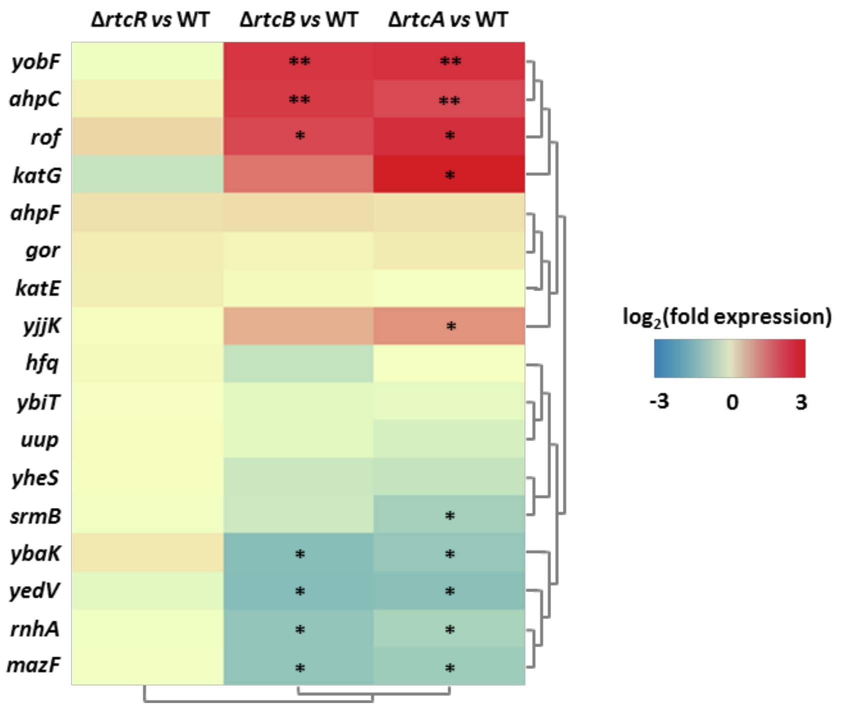

Figure S11. Expression of selected genes in *rtc* gene deletion mutants, Related to Figure 5. Heat map of expression levels of various genes in *E. coli* cells lacking *rtc* compared to the wild-type, as shown by NGS. Deletion or overexpression of these genes is known to induce or repress *rtc* expression levels. Rows and columns have been grouped based on a hierarchical clustering algorithm. Limma * adjusted P-value < 0.05; ** adjusted P-value < 0.01.

Data S3. Identification of potentially cleaved RNAs, Related to Figure 6.

In order to identify potentially cleaved RNAs in transcriptomes, Pearson's correlation coefficient (PCC) of NGS coverage was calculated for each gene individually between datasets derived from both the same and different *E. coli* strains. The method was initially validated on NGS datasets from *E. coli* cells expressing the ribotoxin VapC (Engl *et al.*, 2016): PCC of initiator tRNAs (tRNAfMet) is > 0.9 within VapC-expressing and VapC non-expressing conditions, while it is < 0 when comparing VapC-expressing to VapC non-expressing conditions (Fig. S12A). In contrast, PCC is > 0.9 for elongator tRNAs (tRNAMet) which are not cleaved (Winther & Gerdes, 2011) regardless of VapC expression (Fig. S12A). The majority of genes (over 80%) have a PCC > 0.5 when comparing datasets derived from the same *E. coli* strains: wild-type, Hpx-[RtcON] and Hpx-[RtcOFF] at 8 h post-inoculation (Fig. 6A). Comparison between Hpx-[RtcON] and wild-type revealed a marginally lower correlation but the trend becomes more evident when comparing Hpx-[RtcOFF] to wild-type (Fig. 6A), while a similar distribution was noted at 24 h post-inoculation (Fig. S12D). Transcripts with PCC > 0.5 when comparing datasets of the same strain but < 0.5 when comparing both Hpx-[RtcON] and Hpx-[RtcOFF] to wild-type were considered potentially damaged. Transcripts with PCC > 0.5 when comparing datasets of the same strain and Hpx-[RtcON] to wild-type but < 0.5 when comparing Hpx-[RtcOFF] to wild-type were considered potentially damaged and then repaired by the Rtc system. The cutoff was set to PCC = 0.75 when comparing Δ*gor*, Δ*mazF* or Δ*srmB* to wild-type.

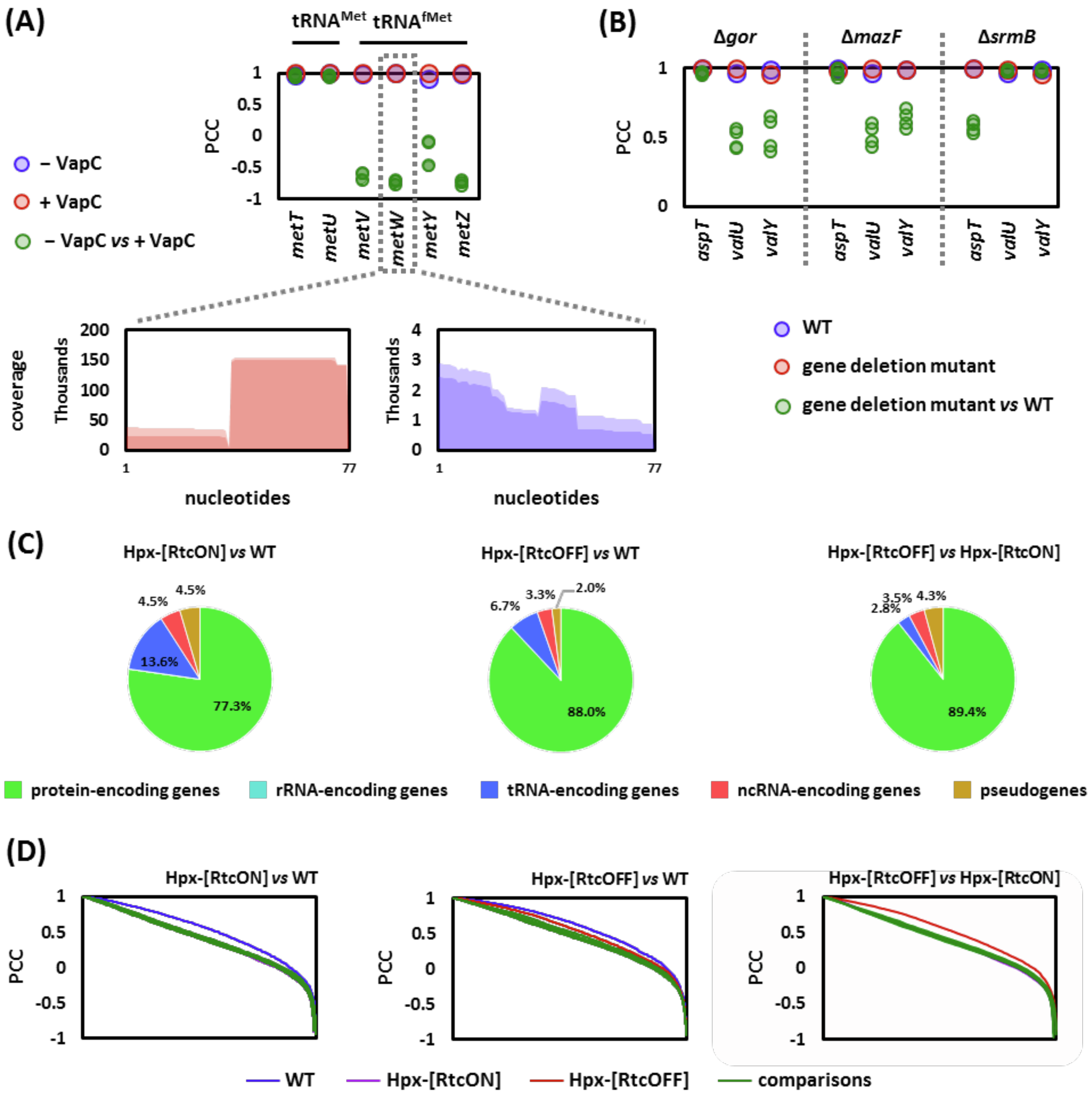

Figure S12. Analysis of damaged RNAs, Related to Figure 6. (A) Initiator but not elognator tRNAs are cleaved by VapC (top) and this is evident using Pearson's Correlation Coefficient (PCC). Distribution of all reads mapped to *metV* in VapC expressing (bottom left) and VapC non-expressing (bottom right) cells reveals the VapC cleavage site. **(B)** Selected tRNAs are damaged in cells lacking *gor*, *mazF* and *srmB* as compared to wild-type. **(C)** The general functional roles of transcripts damaged in Hpx-[RtcON] compared to wild-type (left) and Hpx-[RtcOFF] compared to wild-type (middle) and Hpx-[RtcOFF] compared to Hpx-[RtcON] (right) cells 8 h post-inoculation. **(D)** Transcripts damaged in Hpx-[RtcON] compared to wild-type (left), Hpx-[RtcOFF] compared to wild-type (middle) and Hpx-[RtcOFF] compared to Hpx-[RtcON] (right) cells 24 h post-inoculation, as illustrated by Pearson's Correlation Coefficient (PCC).

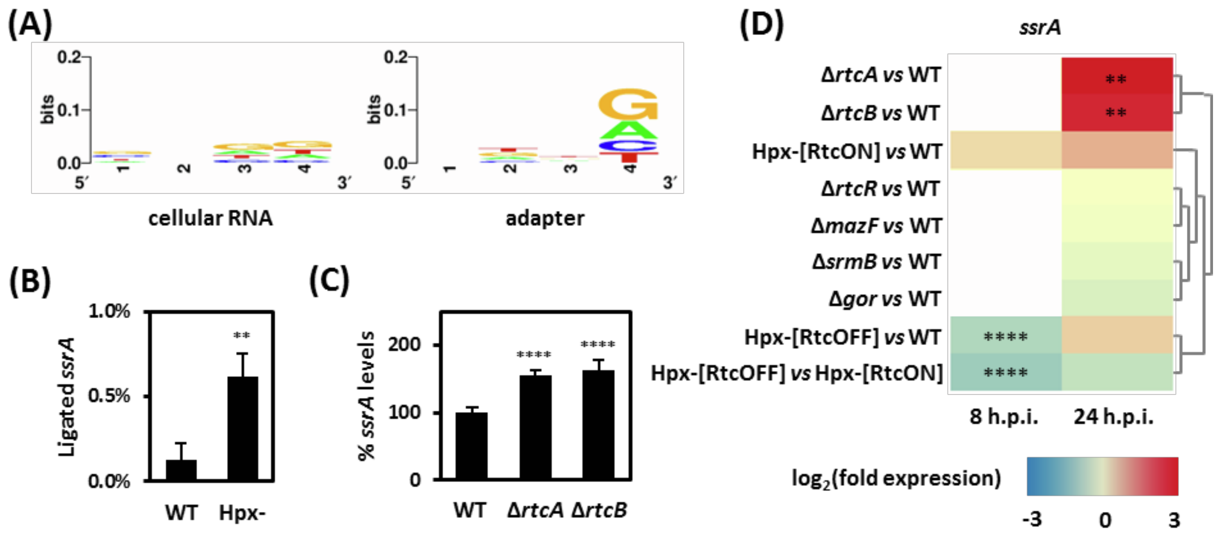

Figure S13. RtcB mediated ligation, Related to Figure 7. (A) Weblogo of 2709 3' termini of cellular RNAs and 5' termini of adapters following RtcB-mediated ligation. Maximum value of the Y-axis for nucleotide sequences is 2.0. **(B)** The tmRNA *ssrA* is targeted more frequently by RtcB ligase in Hpx- compared to the wild-type. **(C)** Expression levels of *ssrA* are upregulated in Δ*rtcA* and Δ*rtcB* compared to the wild-type, as shown by RT-qPCR; mRNA levels of the wild-type strain is set as 100%. Data are shown as mean and error bars represent standard deviation from the mean. *N*=3 and represents total number of independent biological replicates, with 3 technical replicates each. ANOVA ** P-value < 0.01; **** P-value < 0.0001. **(D)** Heat map of *ssrA* expression levels in a range of mutants, as shown by NGS. Rows and columns have been grouped based on a hierarchical clustering algorithm. DESeq2/Limma ** adjusted P-value < 0.01; **** P-value < 0.0001.

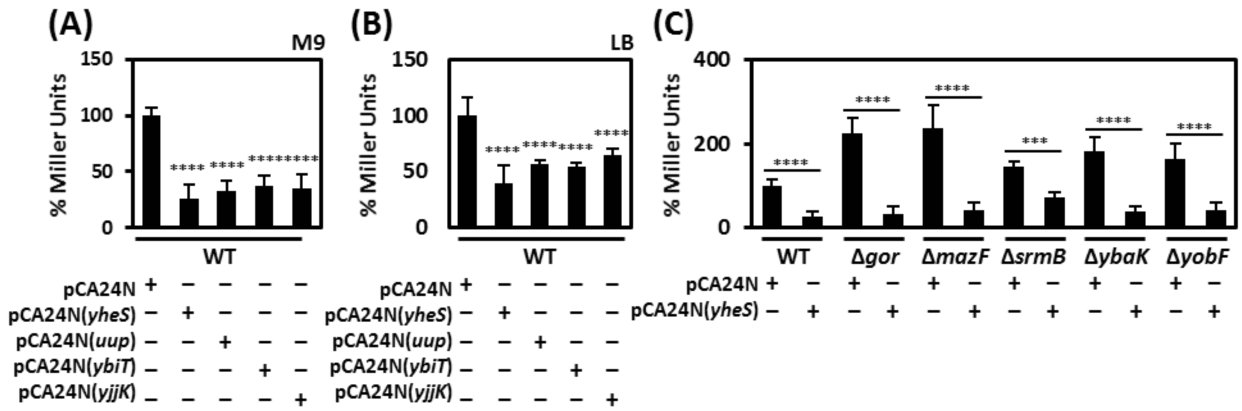

Figure S14. Repression of the Rtc system by gene overexpression, Related to Figure 8. Overexpression of the *yheS* gene and its paralogues, *uup*, *ybiT* and *yjjK* results in repression of the *rtcBA* promoter activity in **(A)** minimal and **(B)** complete medium. **(C)** Overexpression of the YheS protein has an epistatic effect on the gene deletion mutants, resulting in repression the the *rtcBA* promoter activity. Beta-galactosidase activity of the wild-type strain is set as 100%. Data are shown as mean and error bars represent standard deviation from the mean. *N*=3 and represents total number of independent biological replicates, with 3 technical replicates each. ANOVA * P-value < 0.05; ** P-value < 0.01; *** P-value < 0.001; **** P-value < 0.0001.

Table S13. Plasmids, Related to STAR Methods.

Name	Description	Reference
pBBR1MCS-4(P_{rtcBA}-*lacZ*)	Reporter plasmid; produces β-gal controlled by P_{rtcBA}	Engl *et al.*, 2016
pBBR1MCS-4(P_{hrpL}-*lacZ*)	Reporter plasmid; produces β-gal controlled by P_{hrpL}	Jovanovic *et al.*, 2011
pTE103($P_{rtcBA\text{-}nifH}$)	Plasmid containing P_{rtcBA} UAS and P_{nifH} -24/-12 σ^{54} binding site	this study
pBAD18cm(*rtcR*)	Expression plasmid; produces RtcR	Engl *et al.*, 2016
pBAD18cm($rtcR_{\Delta CARF}$)	Expression plasmid; produces RtcR lacking CARF domain	Engl *et al.*, 2016
pBAD18cm(*rtcB*)	Expression plasmid; produces RtcB	Engl *et al.*, 2016
pBAD18cm($rtcB_{H337A}$)	Expression plasmid; produces catalytic mutant $RtcB_{H337A}$	Engl *et al.*, 2016
pBAD18cm(*rtcA*)	Expression plasmid; produces RtcA	Engl *et al.*, 2016
pBAD18cm($rtcA_{H308A}$)	Expression plasmid; produces catalytic mutant $RtcA_{H308A}$	Engl *et al.*, 2016
pMALc2(RtcR)	Expression plasmid; produces MBP-tagged RtcR	this study
pET28($rtcR_{\Delta CARF}$)	Expression plasmid; produces his-tagged $RtcR_{\Delta CARF}$	this study
pET53(*rtcB*)	Expression plasmid; produces his-tagged RtcB	Peach *et al.*, 2015
pET33(*rtcA*)	Expression plasmid; produces his-tagged RtcA	this study
pCA24N(*rof*)	Expression plasmid; produces Rof	ASKA library
pCA24N(*yedV*)	Expression plasmid; produces YedV	ASKA library
pCA24N(*yheS*)	Expression plasmid; produces YheS	ASKA library
pKNT25(*rtcR*)	2-hybrid plasmid; produces T25-RtcR	this study
pKNT25(*rtcB*)	2-hybrid plasmid; produces T25-RtcB	this study
pKNT25(*rtcA*)	2-hybrid plasmid; produces T25-RtcA	this study
pKNT25($rtcR_{1\text{-}186}$)	2-hybrid plasmid; produces T25-$RtcR_{CARF}$	this study
pKNT25($rtcR_{1\text{-}353}$)	2-hybrid plasmid; produces T25-$RtcR_{CARF\text{-}AAA}$	this study
pKNT25($rtcR_{187\text{-}353}$)	2-hybrid plasmid; produces T25-$RtcR_{AAA}$	this study
pKNT25($rtcR_{187\text{-}532}$)	2-hybrid plasmid; produces T25-$RtcR_{AAA\text{-}HTH}$	this study
pKNT25($rtcR_{354\text{-}532}$)	2-hybrid plasmid; produces T25-$RtcR_{HTH}$	this study
pKT25(*rtcR*)	2-hybrid plasmid; produces RtcR-T25	this study
pKT25(*rtcB*)	2-hybrid plasmid; produces RtcB-T25	this study
pKT25(*rtcA*)	2-hybrid plasmid; produces RtcA-T25	this study
pKT25($rtcR_{1\text{-}186}$)	2-hybrid plasmid; produces $RtcR_{CARF}$-T25	this study
pKT25($rtcR_{1\text{-}353}$)	2-hybrid plasmid; produces $RtcR_{CARF\text{-}AAA}$-T25	this study
pKT25($rtcR_{187\text{-}353}$)	2-hybrid plasmid; produces $RtcR_{AAA}$-T25	this study
pKT25($rtcR_{187\text{-}532}$)	2-hybrid plasmid; produces $RtcR_{AAA\text{-}HTH}$-T25	this study
pKT25($rtcR_{354\text{-}532}$)	2-hybrid plasmid; produces $RtcR_{HTH}$-T25	this study
pKT25-zip	2-hybrid plasmid; positive control	BACTH System Kit
pUT18(*rtcR*)	2-hybrid plasmid; produces T18-RtcR	this study
pUT18(*rtcB*)	2-hybrid plasmid; produces T18-RtcB	this study
pUT18(*rtcA*)	2-hybrid plasmid; produces T18-RtcA	this study
pUT18($rtcR_{1\text{-}186}$)	2-hybrid plasmid; produces T18-$RtcR_{CARF}$	this study
pUT18($rtcR_{1\text{-}353}$)	2-hybrid plasmid; produces T18-$RtcR_{CARF\text{-}AAA}$	this study
pUT18($rtcR_{187\text{-}353}$)	2-hybrid plasmid; produces T18-$RtcR_{AAA}$	this study
pUT18($rtcR_{187\text{-}532}$)	2-hybrid plasmid; produces T18-$RtcR_{AAA\text{-}HTH}$	this study
pUT18($rtcR_{354\text{-}532}$)	2-hybrid plasmid; produces T18-$RtcR_{HTH}$	this study
pUT18C(*rtcR*)	2-hybrid plasmid; produces RtcR-T18	this study
pUT18C(*rtcB*)	2-hybrid plasmid; produces RtcB-T18	this study
pUT18C(*rtcA*)	2-hybrid plasmid; produces RtcA-T18	this study
pUT18C($rtcR_{1\text{-}186}$)	2-hybrid plasmid; produces $RtcR_{CARF}$-T18	this study
pUT18C($rtcR_{1\text{-}353}$)	2-hybrid plasmid; produces $RtcR_{CARF\text{-}AAA}$-T18	this study
pUT18C($rtcR_{187\text{-}353}$)	2-hybrid plasmid; produces $RtcR_{AAA}$-T18	this study
pUT18C($rtcR_{187\text{-}532}$)	2-hybrid plasmid; produces $RtcR_{AAA\text{-}HTH}$-T18	this study
pUT18C($rtcR_{354\text{-}532}$)	2-hybrid plasmid; produces $RtcR_{HTH}$-T218	this study
pUT18C-zip	2-hybrid plasmid; positive control	BACTH System Kit
pKT25(*hrpS*)	2-hybrid plasmid; produces T25-HrpS	Jovanovic *et al.*, 2014
pKT25(*hrpV*)	2-hybrid plasmid; produces T25-HrpV	Jovanovic *et al.*, 2014
pUT18C(*hrpS*)	2-hybrid plasmid; produces T18-HrpS	Jovanovic *et al.*, 2014
pUT18C(*hrpV*)	2-hybrid plasmid; produces T18-HrpV	Jovanovic *et al.*, 2014
pBBR-fusA-lacZa	pBBR-MCS5 encoding P_{tet}-lacZw-T1, P_{lac}-LacZa-T1, lacI; reporter plasmid for translation elongation speed	A gift from Y. Wang; Zhu *et al.*, 2016
pSG25	P_{tacI}-lacZ wildtype; reporter plasmid for translation fidelity	Addgene #63867; O'Connor *et al.*, 1993
pSGlacZ	P_{tacI}-lacZ +1 frameshift; reporter plasmid for translation fidelity	Addgene #63874; O'Connor *et al.*, 1993
pSG12DP	P_{tacI}-lacZ -1 frameshift; reporter plasmid for translation fidelity	Addgene #63876; O'Connor *et al.*, 1993
pSG34-11	P_{tacI}-lacZ premature UGA stop; reporter plasmid for translation fidelity	Addgene #63869; O'Connor *et al.*, 1993
pSG12-6	P_{tacI}-lacZ premature UAG stop; reporter plasmid for translation fidelity	Addgene #63870; O'Connor *et al.*, 1993
pSG853	P_{tacI}-lacZ premature UAA stop; reporter plasmid for translation fidelity	Addgene #63873; O'Connor *et al.*, 1993

Table S14. Bacterial strains, Related to STAR Methods.

Name	Description	Reference
MG1655	*E. coli* K-12, wild-type	
MG1655 Δ*rtcR*	MG1655 strain lacking *rtcR*	Engl *et al.*, 2016
MG1655 Δ*rtcB*	MG1655 strain lacking *rtcB*	Engl *et al.*, 2016
MG1655 Δ*rtcA*	MG1655 strain lacking *rtcA*	Engl *et al.*, 2016
MG1655 Δ*gor*	MG1655 strain lacking *gor*	Engl *et al.*, 2016
MG1655 Δ*gor* Δ*rtcR*	MG1655 strain lacking *gor* and *rtcR*	this study
MG1655 Δ*gor* Δ*rtcB*	MG1655 strain lacking *gor* and *rtcB*	this study
MG1655 Δ*gor* Δ*rtcA*	MG1655 strain lacking *gor* and *rtcA*	this study
MG1655 Δ*hfq*	MG1655 strain lacking *hfq*	this study
MG1655 Δ*hfq* Δ*rtcR*	MG1655 strain lacking *hfq* and *rtcR*	this study
MG1655 Δ*hfqF* Δ*rtcB*	MG1655 strain lacking *hfq* and *rtcB*	this study
MG1655 Δ*hfqF* Δ*rtcA*	MG1655 strain lacking *hfq* and *rtcA*	this study
MG1655 Δ*mazF*	MG1655 strain lacking *mazF*	this study
MG1655 Δ*mazF* Δ*rtcR*	MG1655 strain lacking *mazF* and *rtcR*	this study
MG1655 Δ*mazF* Δ*rtcB*	MG1655 strain lacking *mazF* and *rtcB*	this study
MG1655 Δ*mazF* Δ*rtcA*	MG1655 strain lacking *mazF* and *rtcA*	this study
MG1655 Δ*srmB*	MG1655 strain lacking *srmB*	this study
MG1655 Δ*rnhA*	MG1655 strain lacking *rnhA*	this study
MG1655 Δ*rnhA* Δ*rtcR*	MG1655 strain lacking *rnhA* and *rtcR*	this study
MG1655 Δ*rnhA* Δ*rtcB*	MG1655 strain lacking *rnhA* and *rtcB*	this study
MG1655 Δ*rnhA* Δ*rtcA*	MG1655 strain lacking *rnhA* and *rtcA*	this study
MG1655 Δ*rppH*	MG1655 strain lacking *rppH*	this study
MG1655 Δ*rppH* Δ*rtcR*	MG1655 strain lacking *rppH* and *rtcR*	this study
MG1655 Δ*rppH* Δ*rtcB*	MG1655 strain lacking *rppH* and *rtcB*	this study
MG1655 Δ*rppH* Δ*rtcA*	MG1655 strain lacking *rppH* and *rtcA*	this study
MG1655 Δ*ybaK*	MG1655 strain lacking *ybaK*	Engl *et al.*, 2016
MG1655 Δ*ybaK* Δ*rtcR*	MG1655 strain lacking *ybaK* and *rtcR*	this study
MG1655 Δ*ybaK* Δ*rtcB*	MG1655 strain lacking *ybaK* and *rtcB*	this study
MG1655 Δ*ybaK* Δ*rtcA*	MG1655 strain lacking *ybaK* and *rtcA*	this study
MG1655 Δ*yobF*	MG1655 strain lacking *yobF*	Engl *et al.*, 2016
MG1655 Δ*yobF* Δ*rtcR*	MG1655 strain lacking *yobF* and *rtcR*	this study
MG1655 Δ*yobF* Δ*rtcB*	MG1655 strain lacking *yobF* and *rtcB*	this study
MG1655 Δ*yobF* Δ*rtcA*	MG1655 strain lacking *yobF* and *rtcA*	this study
Hpx-	MG1655 strain lacking *katE*, *katG* and *ahpC*	Park *et al.*, 2005
MG1655 Δ*ahpC*	MG1655 strain lacking *ahpC*	this study
MG1655 Δ*ahpC* Δ*rtcR*	MG1655 strain lacking *ahpC* and *rtcR*	this study
MG1655 Δ*ahpC* Δ*rtcB*	MG1655 strain lacking *ahpC* and *rtcB*	this study
MG1655 Δ*ahpC* Δ*rtcA*	MG1655 strain lacking *ahpC* and *rtcA*	this study
MG1655 Δ*ahpF*	MG1655 strain lacking *ahpF*	this study
MG1655 Δ*katE*	MG1655 strain lacking *katE*	this study
MG1655 Δ*katG*	MG1655 strain lacking *katG*	this study
MG1655 Δ*fur*	MG1655 strain lacking *fur*	this study
MG1655 Δ*fur* Δ*rtcB*	MG1655 strain lacking *fur* and *rtcB*	this study
MG1655 Δ*iscR*	MG1655 strain lacking *iscR*	this study
MG1655 Δ*iscR* Δ*rtcB*	MG1655 strain lacking *iscR* and *rtcB*	this study
MG1655 Δ*oxyR*	MG1655 strain lacking *oxyR*	this study
MG1655 Δ*oxyR*Δ*rtcB*	MG1655 strain lacking *oxyR* and *rtcB*	this study
